# Microbial mechanisms underlying the shift of net global warming potential in a maize field under organic amendments

**DOI:** 10.3389/fmicb.2026.1966738

**Published:** 2026-10-22

**Authors:** Daijia Fan, Wentao Xue, Ling Zhang, Liang He, Guoyuan Zou, Xingjian Zhang, Xiaohang Zhao, Chunxiao Han, Wentian He

**Affiliations:** 1 Institute of Plant Nutrition, Resources and Environment, Beijing Academy of Agriculture and Forestry Sciences, Beijing, China; 2 Beijing Engineering Technology Research Center for Slow/Controlled-Release Fertilizer, Beijing, China; 3 National Meteorological Centre, Beijing, China; 4 Beijing Bihaiyuan Ecological Agriculture Sightseeing Co., Ltd., Beijing, China; 5 SDIC Xinjiang Luobupo Postash Co., Ltd., Hami, China

**Keywords:** manure application, N_2_O emission, SOC sequestration, soil functional genes, straw return, wheat-maize rotation

## Abstract

While organic amendments are promoted for enhancing soil quality, their impacts on the net global warming potential (GWP) and the underlying microbial mechanisms in the maize system remain elusive. By using a long-term field experiment established in 2007 under a winter wheat-summer maize rotation system, a field study was conducted to investigate the effects of different organic-amended treatments (manure application and straw return coupled with conventional or no tillage) on greenhouse gas (i.e., nitrous oxide [N_2_O] and carbon dioxide [CO_2_]) emissions, soil organic carbon (SOC) stocks and functional genes governing soil C and N cycling during the 2021 and 2022 maize growing seasons. Compared to conventional N fertilization (CN), long-term organic amendments, particularly manure application, significantly enhanced SOC stocks by 16.7–46.2% while reducing cumulative N_2_O emissions by 22.9–30.2%, respectively. Although a 22.6–44.5% increase in CO_2_ emissions were observed under organic amendments relative to CN, the concurrent SOC gains and N_2_O mitigation shifted the field-scale net GWP from positive to negative. Multivariate analyses implied that enhanced SOC sequestration under organic amendments was mainly associated with greater exogenous organic matter inputs and enriched C fixation genes, and lower N_2_O emissions were accompanied by lower bacterial *amoA* and higher *nosZ* gene abundances, suggesting that reduced nitrification and enhanced N_2_O reduction potential may have contributed to N_2_O mitigation. Therefore, long-term manure application is a viable strategy to achieve climate-smart maize production by enhancing SOC sequestration, mitigating N_2_O emissions and maintaining yield without additional chemical N input.

## 1 Introduction

As the breadbasket of China, the North China Plain (NCP) covers 24% of the total cropping area and produces 60 and 31% of the total wheat and maize in China, respectively (National Bureau of Statistics of China, 2024). Winter wheat (*Triticum aestivum* L.)-summer maize (*Zea mays* L.) rotation is the dominant cropping system of this region (Li et al., 2020). However, excessive and inappropriate chemical nitrogen (N) fertilization has been widely adopted by local farmers to obtain high yields, thus leading to low N use efficiency, high greenhouse gas (GHG) emissions and decreased soil organic carbon (SOC) stocks (Tan et al., 2017; Gai et al., 2018). Optimizing fertilization practices to attenuate GHG emissions and improve SOC stock while maintaining crop yields has become a major challenge for the sustainable agricultural production in this region (Chen et al., 2014).

Organic amendments such as straw return and the application of poultry manure can maintain soil fertility and quality mainly by providing a balanced and sustained supply of nutrients, improving soil aggregate stability and decreasing soil bulk density (Bhattacharyya et al., 2007; Diacono and Montemurro, 2011). In addition, organic amendments are considered an effective approach for increasing SOC sequestration via direct exogenous organic matter inputs (Dignac et al., 2017; Dai et al., 2019). Previous meta-analyses reported that organic amendments led to a 20–90% increase in SOC stocks in the topsoil over periods ranging from 3 to 82 years (Diacono and Montemurro, 2011; Maillard and Angers, 2014). Maize field experiments previously conducted in NCP also suggested that organic amendments enhanced SOC storage and converted the maize fields from net GHG sources to GHG sinks (Li et al., 2018; Chang et al., 2020). However, the organic amendments often contain a large quantity of readily decomposable N-rich compounds, thus inducing a greater risk of nitrous oxide (N_2_O) emissions (Cayuela et al., 2010). Some studies found that the application of organic manure or crop straw increased the N_2_O emissions from the maize fields in NCP (Zhang et al., 2012; Hu et al., 2013), while Lv et al. (2020) observed that the addition of organic manure had lower N_2_O emission than traditional fertilization under wheat-maize rotation. These inconsistent conclusions were attributed to the wide variations in soil properties (e.g., texture, moisture and SOC) and climatic conditions (Charles et al., 2017). It was reported that the crop residue addition-induced N_2_O emissions were greater in fine-textured soils than that in coarse-textured soils (Chen et al., 2013). Moreover, Gu et al. (2017) revealed that 88–151% of the sequestered C would be offset by high N_2_O emissions at saturated SOC levels under the application of dairy manure or crop residues in a wheat-maize cropping system in China. Thus, it is essential to reveal the underlying mechanisms of the highly site-specific impacts of organic amendments on the net global warming potential (GWP) in the wheat-maize rotation system in NCP.

Soil microbial communities have been known to play a pivotal role in SOC dynamics (Liang et al., 2017). Agricultural management practices such as organic fertilization and straw return have significant effects on the abundance, structure, and diversity of microbial communities (Zhang et al., 2018; Cong et al., 2020; Liu et al., 2022). Wu et al. (2021) and Bei et al. (2022) found that the addition of external organic materials such as manure and crop residues enhanced SOC decomposition due to the increased microbial biomass, diversity and activity in soil. Inconsistently, Zhao et al. (2019) found that straw addition decreased soil microbial richness and diversity, while Hupfauf et al. (2016) observed no significant variations in soil microbial communities following organic amendments. Moreover, numerous studies reported that GHG emissions were significantly regulated by organic fertilization and straw return by altering the abundance and structure of functional gene harboring microbial communities such as nitrifiers and denitrifiers (Liu et al., 2019). Huang et al. (2019) reported that straw return increased the N_2_O emissions from a wheat-maize rotation system due to the increased N_2_O production via enhanced ammonia oxidizing bacteria and reduced N_2_O consumption via decreased *nosZ*-harboring denitrifiers. Hu et al. (2022) revealed that manure application could enhance N_2_O emission potential resulted from promoted denitrification in soil. In contrast, the studies by Zhou et al. (2017) and Duan et al. (2019) suggested that straw return and manure application reduced the N_2_O emissions via decreased denitrification under high soil moisture conditions. In addition, long-term organic amendments may lead to changes in soil pH, nutrient availability and subsequently soil microbes, and ultimately affect the sequestration and mineralization of SOC as well as the generation of GHGs (Linton et al., 2020; Bhattacharyya et al., 2022). These inconsistent effects derive from the sophisticated interplays among organic materials, soil microbial communities and soil abiotic factors in various cropping systems. Therefore, further evaluation of the differential responses of key functional microbial communities involved in SOC sequestration and GHG emissions to organic amendments is required for the sustainable production of wheat-maize rotation system.

In this study, we hypothesized that organic amendments could decrease the net GWP by synergistically improving SOC sequestration and mitigating N_2_O emissions mainly via the regulation of key functional microbial communities in the wheat-maize rotation system in NCP. Thus, a field study was conducted during 2021 and 2022 based on a long-term field experiment established in 2007 in the suburb of Beijing, aiming to (i) determine the effects of different organic-amended treatments including manure application and straw return on crop yield, GHG emissions, SOC sequestration and the abundance and composition of related microbial functional genes, and (ii) elucidate the underlying microbial regulatory mechanisms.

## 2 Materials and methods

### 2.1 Study site

The field experiment was conducted in Shilou County, Fangshan District, Beijing in NCP (116°08′E, 39°40′N), which has a temperate continental monsoon climate with an average annual air temperature of 13.2 °C and an average annual precipitation of 670.4 mm. Based on the Chinese Soil Taxonomy, the soil is classified as a clay loam cinnamon fluvo-aquic soil, similar to Eutric Cambisol in the FAO soil classification system (IUSS Working Group WRB, 2022). The properties of topsoil (0–20 cm) prior to the experiment were as follows: organic matter 12.6 g kg^−1^, total N 0.75 g kg^−1^, available phosphorus (P) 6.04 mg kg^−1^, available potassium (K) 56.4 mg kg^−1^, bulk density 1.25 g cm^−3^, and pH 8.21.

### 2.2 Experimental design and field management

Seven N fertilization treatments were included in this experiment: (1) CK: no N fertilizer; (2) CN: conventional urea (46% N) applied at 180 kg N ha^−1^; (3) CNM: 75% urea N plus 25% composted poultry manure N; (4) CNS: 75% urea N plus 25% composted slurry N; (5) CM: 100% composted poultry manure N; (6) CT: 75% urea N plus 25% straw N (straw return with deep tillage); (7) NT: 75% urea N plus 25% straw N (straw return with no tillage). All N fertilization treatments were applied at the same N rate. Same fertilization treatments have been conducted for both the wheat and maize growing seasons ever since 2007.

All treatments followed a randomized complete block design with three replicates per treatment. The area of each plot was 80 m^2^ (10 m × 8 m). The soil of all plots was rotovated to a depth of 15–20 cm before sowing by a rotary tiller except for those under NT treatment. Winter wheat was sown in mid-October and harvested in early June of the following year, and summer maize was sown in late June and harvested in early October.

All N-fertilized treatments received the same total N, P and K inputs at rates of 180 kg N ha^−1^ season^−1^, 95 kg P ha^−1^ season^−1^ and 90 kg K ha^−1^ season^−1^, respectively. For CN, CNM, CNS, CT and NT treatments, two thirds of the total chemical N as well as all chemical P and K fertilizers were applied as basal fertilizer in the form of compound fertilizer (N-P-K formulations adjusted accordingly to achieve consistent total N, P and K inputs across different fertilized treatments), with rest of the chemical N applied as top-dressing fertilizer in the form of conventional urea (46% N) at the jointing stage of wheat or maize. For CNM, CNS and CM treatments, manure or slurry was applied all at once as basal fertilizer. Wheat and maize straw was pulverized and returned to the soil immediately after harvest under CT and NT treatments, whereas all straw was removed under the other treatments. Basal fertilizers were homogeneously incorporated into soil before sowing, whereas the top-dressing fertilizers were surface broadcast by hand. Wheat and maize were subjected to the same fertilization regime. The chemical properties of composted poultry manure and slurry are listed in Table 1. The seasonal total C, N, P and K inputs under different fertilization treatments are shown in Table 2.

**Table 1:** Chemical properties of composted poultry manure and slurry.

Organic fertilizer	Total N	Total P	Total K	Organic matter	pH
Poultry manure	2.86%	3.35%	2.91%	39.1%	7.85
Slurry	2.88%	5.03%	0.67%	40.4%	7.78

**Table 2:** Seasonal total C, N, P, and K inputs under different fertilization treatments during the maize growing season.

Treatment	Total C inputs (kg C ha^−1^)	Total N inputs (kg N ha^−1^)	Total P inputs (kg P ha^−1^)	Total K inputs (kg K ha^−1^)
CK	0	0	0	0
CN	0	180	95	90
CNM	338	180	95	90
CNS	366	180	95	90
CM	1,355	180	95	90
CT	2,728	180	95	90
NT	2,728	180	95	90

### 2.3 Sampling and chemical analysis

This study mainly focused on the maize growing seasons in 2021 and 2022, the daily air temperature and precipitation during which are shown in Supplementary Figure 1. Five maize plants were randomly collected from each plot at the harvest stage and weighed for yield. The N content of maize plant was determined using the Kjeldahl digestion method (Suhre et al., 1982).

Five soil samples (0–20 cm) were randomly collected from each plot at the harvest stage of maize using soil augers (4.5 cm inner diameter) and homogeneously mixed into a composite sample. One subsample of each fresh composite sample was immediately extracted with 1.0 M KCl solution and measured for ammonium N (NH_4_^+^–N) and nitrate N (NO_3_^−^–N) contents using a continuous flow injection analyzer (Seal-AA3, Shanghai, China) (McDaniel et al., 2020), a second was air-dried and sieved (2 mm) for chemical analysis, and the remaining subsample was stored at −20 °C for subsequent microbial analysis. Soil pH was measured by a pH meter by adding deionized water into sieved soil with a soil-to-water ratio of 1:5 (w/v). The contents of SOC and soil total N were determined by dry combustion using an elemental analyzer (FlashSmart NC SOIL, Beijing, China; Roper et al., 2019). Soil bulk density was measured with the cutting ring method (Xie et al., 2020). Soil moisture was measured using a portable soil moisture sensor (Acclima RD1200, Idaho, USA) at 10 cm soil depth.

The SOC stock was calculated using the fixed depth method as shown in Equation 1 (Bhattacharyya et al., 2011):

$$\text{SOCD}=\mathrm{SOC}\times \mathrm{BD}\times D\times 10^{–1}$$ (1)

Where *SOCD* is SOC stock of 0–20 cm soil layer (t ha^−1^), *SOC* is SOC content (g kg^−1^), *BD* is bulk density (g cm^−3^), and *D* is soil layer depth (20 cm). The rate of SOC sequestration induced by N fertilization relative to unfertilized control was calculated using Equation 2 (Ghosh et al., 2018; Wu et al., 2024):

$$\mathrm{SO}C_{\mathrm{SR}}=(\mathrm{SOC}D_{T}-\mathrm{SOC}D_{\mathrm{CK}})/n$$ (2)

Where *SOC_SR_* is SOC sequestration rate of N-fertilized treatment over unfertilized treatment (t ha^−1^ yr.^−1^), *SOCD_T_* and *SOCD_CK_* are SOC stocks under N-fertilized and unfertilized treatments (t ha^−1^), respectively, and *n* is the cumulative number of years of external organic C input (yr). The seasonal SOC sequestration efficiency of N-fertilized treatment relative to unfertilized treatment was obtained according to Equations 3, 4 (Jiang et al., 2014; Cai et al., 2015):

$$\mathrm{SO}C_{\mathrm{SE}}=(\mathrm{SOC}D_{T}-\mathrm{SOC}D_{\mathrm{CK}})/(C_{\text{input}-T}-C_{\text{input}-\mathrm{CK}})$$ (3)

$$C_{\text{input}}=(Y_{g}+Y_{s})\times R\times \mathrm{Tr}\times C_{\text{crop}}/10^{6}+DM_{\mathrm{OA}}\times C_{\mathrm{OA}}/10^{3}$$ (4)

Where *SOC_SE_* is the SOC sequestration efficiency (%), *C_input-T_* and *C_input-CK_* denote the external organic C inputs into soil under N-fertilized and unfertilized treatments during the maize growing seasons (t ha^−1^), respectively, which mainly originated from crop roots and organic amendments. *Y_g_* is the dry mass of maize grain yield (kg ha^−1^), *Y_s_* is the dry mass of maize straw yield (kg ha^−1^), *R* is the ratio of maize root C to aboveground C (26% for maize), *Tr* is the proportion of root biomass distributed within the topsoil layer (0–20 cm; 85% for maize), *C_crop_* is the C content of the aboveground parts (g kg^−1^; 427 g kg^−1^ for maize), *DM_OA_* is the dry mass of straw or manure returned to the soil under organic-amended treatments (t ha^−1^), and *C_OA_* is the C content of straw or manure returned to the soil under organic-amended treatments (g kg^−1^). Root exudates, which typically account for 5–10% of net photosynthate (Haller and Stolp, 1985), were not measured and are therefore not included in *C_input_*. This may lead to a slight overestimation of SOC sequestration efficiency.

Soil N_2_O and carbon dioxide (CO_2_) emissions were determined by static chamber-gas chromatography method (Ye et al., 2020). Column chambers (34.5 cm diameter and 60 cm high) made of polyvinyl chloride (PVC) plastic, which was installed with a digital thermometer for temperature monitoring and an electric fan for air mixing, were used for gas sampling. Two chamber bases were inserted 7–8 cm deep into the soil in the crop interrows of each plot after sowing. Gas samples were collected between 9:00 a.m. and 11:00 a.m. daily after every N fertilization or rainfall event for 3–5 days or once every 3–10 days otherwise. Four gas samples were successively collected from each plot at the interval of 10 min (0, 10, 20, and 30 min) and were measured for GHG concentration via a gas chromatography (Shimadzu, GC-14 B, Japan). The GHG flux was calculated by Equation 5 (Fan et al., 2022):

F=ρ×H×(Δc/Δt)×273/(273+T) (5)

Where *F* is the GHG flux (mg m^−2^ h^−1^), *ρ* is the standard gas density (mg m^−3^), *H* is the chamber headspace height (m), *Δc*/*Δt* is the increasing rate of gas concentration in the chamber (mg m^−3^ h^−1^), and *T* is the mean temperature in the chamber during gas sampling (°C). The seasonal cumulative GHG emissions were calculated according to Fan et al. (2022). The global warming potential (GWP) was computed using Equation 6 (Qi et al., 2020):

$$\mathrm{GWP}=(\mathrm{SE}-CO_{2}+\mathrm{SE}-N_{2}O\times 273)/1000$$ (6)

Where *GWP* is the GWP of maize field (t CO_2_-eq ha^−1^), *SE-CO_2_* and *SE-N_2_O* are the seasonal cumulative emissions of CO_2_ (kg CO_2_ ha^−1^) and N_2_O (kg N_2_O ha^−1^), and 273 is the 100-year time horizon GWP value of N_2_O relative to CO_2_.

The seasonal net GWP (t CO_2_-eq ha^−1^) was determined by Equation 7 (IPCC, 2013):

$$\mathrm{net}\;\mathrm{GWP}=-\text{dSOC}\times 44/12+\mathrm{SE}-N_{2}O\times 273/1000$$ (7)

Where *dSOC* is the rate of change in SOC stocks during the maize growing season (t C ha^−1^).

### 2.4 DNA extraction and microbial analysis

The genomic DNA from soil was extracted and purified using Fast DNA SPIN Kit (K201, Bocai, Shanghai) according to the manufacturer’s instructions. The absolute abundance of critical C and N cycling genes was quantified using a high-throughput quantitative-PCR-based chip (target genes and primer sequences listed in Supplementary Table 1). First, Sample Sourceplate and Assy Sourceplate were prepared by adding DNA samples and corresponding primers, respectively, into qPCR reagents. Then, the amplification was conducted in a 100 nL reaction mixture using the SmartChip Real-Time PCR System (Wafergen, Fremont, CA) and target genes were detected and quantified through SmartChip qPCR Software (v. 2.7.0.1) and Canco software (v. 5.0) following the procedures as described by Li et al. (2023).

### 2.5 Statistical analysis

Statistical analysis was performed in R (v. 4.6.1; R Core Team, 2026). For each response variable, a linear mixed-effects model was fitted by restricted maximum likelihood using the “nlme” package (Pinheiro et al., 2026). Fertilization treatment, year (2021 and 2022) and their interaction were included as fixed effects, whereas block (three replicate blocks) and plot within block were included as random intercepts (response ~ Treatment × Year, random = ~1 | Block/Plot). All models were fitted assuming Gaussian residual errors, and no response variables were transformed. Model assumptions were evaluated using normalized residuals, with normality assessed by the Shapiro–Wilk test and homogeneity of variance among Treatment × Year combinations assessed by the Fligner–Killeen test. Fixed effects were evaluated using marginal F tests, and estimated marginal means were calculated from the fixed-effect estimates and their variance–covariance matrix. Pairwise treatment contrasts were adjusted using Tukey’s method based on the studentized range distribution. When the Treatment × Year interaction was significant, treatment simple effects were compared within each year. Otherwise, treatments were compared using marginal means averaged across two years. Fixed effects were considered significant at *p* ≤ 0.05, and pairwise treatment differences at Tukey-adjusted *p* ≤ 0.05.

To account for multiple testing across the functional gene dataset, *p*-values from the gene-level treatment tests and pairwise contrasts against CN (330 contrasts across 55 gene units) were additionally adjusted using the Benjamini–Hochberg false-discovery-rate (FDR) procedure (Benjamini and Hochberg, 1995). Gene-level comparisons were evaluated at FDR-adjusted *p* ≤ 0.05. The microbial functional potential in soil C and N biogeochemical cycling was assessed by quantitative microbial element cycling based on the high-throughput quantitative-PCR chip (Zheng et al., 2018). The soil microbial community assemblage under different treatments was determined by non-metric multidimensional scaling (NMDS) analysis with Bray–Curtis dissimilarity distances using the “vegan” package in R, and the correlations among soil microbial community assemblage and soil properties were identified by distance-based redundancy analysis (db-RDA) and Mantel test using the “ggvegan” and “vegan” packages in R, respectively (Borcard et al., 2018).

Partial least squares-path modeling (PLS-PM) analysis was employed to elaborate the interplays among N fertilization, soil physicochemical properties, soil functional microbial communities, SOC sequestration and GHG emissions using the “plspm” package in R (Sanchez et al., 2022). The analysis included 21 field plots (seven treatments × three blocks, one observation per plot). Based on the hypothesis of this study and priori theoretical knowledge, a conceptual path model was constructed hypothesizing that (i) N fertilization (a categorical observed variable representing the seven fertilization treatments) directly altered soil physicochemical properties (i.e., soil climate [temperature and water content], pH, total N [TN], electrical conductivity [EC] and mineral N [NH_4_^+^–N and NO_3_^−^–N]), (ii) soil physicochemical properties directly shaped the C fixation, C degradation and N cycling gene profiles (as shown in Supplementary Table 1), and (iii) N fertilization, soil properties and functional gene profiles jointly regulated SOC, N_2_O emissions and CO_2_ emissions. All indicators were standardized for estimation, and gene abundances were log_10_-transformed. Mode A weights and the centroid scheme were used, with a convergence tolerance of 10^−6^ and a maximum of 100 iterations. Uncertainty was assessed using 10,000 nonparametric bootstrap resamples, each comprising 21 complete plot records sampled with replacement from the full dataset. Indicator standardization, outer weights, construct scores and structural coefficients were re-estimated in each resample, and construct orientations were aligned with the original-sample loading vectors before summarizing the bootstrap distributions. Pointwise percentile 95% confidence intervals were calculated for path coefficients. Model assessment included outer loadings, cross-loadings, construct correlations, variance inflation factors for each structural equation, R^2^ and adjusted R^2^, and the goodness-of-fit index. For multi-indicator constructs, Cronbach’s alpha, Dillon-Goldstein’s rho and average variance extracted were additionally examined, and heterotrait–monotrait ratios were computed as supplementary diagnostics. Path stability was examined using 21 leave-one-plot-out and three leave-one-block-out refits. Conditional prediction of SOC, N_2_O and CO_2_ used the observed indicators of their immediate predictor constructs, with standardization parameters, outer weights and structural coefficients estimated exclusively from the training data. Prediction errors were summarized as root mean squared error and mean absolute error and benchmarked against predictions based on the overall mean and treatment-specific means of the training sample. Together, these checks evaluated the internal stability and conditional predictive performance of the PLS-PM model.

## 3 Results

### 3.1 Soil climate and available N contents

The soil moisture under CK, CN, CNM, CNS, CM, CT and NT treatments lied between 7.6–32.9%, 6.7–32.3%, 6.9–31.3%, 6.3–32.6%, 6.7–33.0%, 6.8–33.8% and 7.9–33.3%, respectively. The average soil moisture in 2021 was higher than that in 2022 regardless of treatments. Additionally, CNS, CM, CT and NT had higher average soil moisture than CN treatment in both years, with the highest one observed under CT (25.1% in 2021 and 24.7% in 2022) (Figures 1A,B). The soil temperature of CK, CN, CNM, CNS, CM, CT and NT treatments lied between 15.4–37.2, 21.6–38.7, 19.9–38.7, 20.1–38.0, 17.7–37.3, 21.4–37.3 and 19.7–37.9 °C, respectively. In comparison, soil temperature generally remained the same despite treatments (Figures 1C,D).

**Figure 1:**
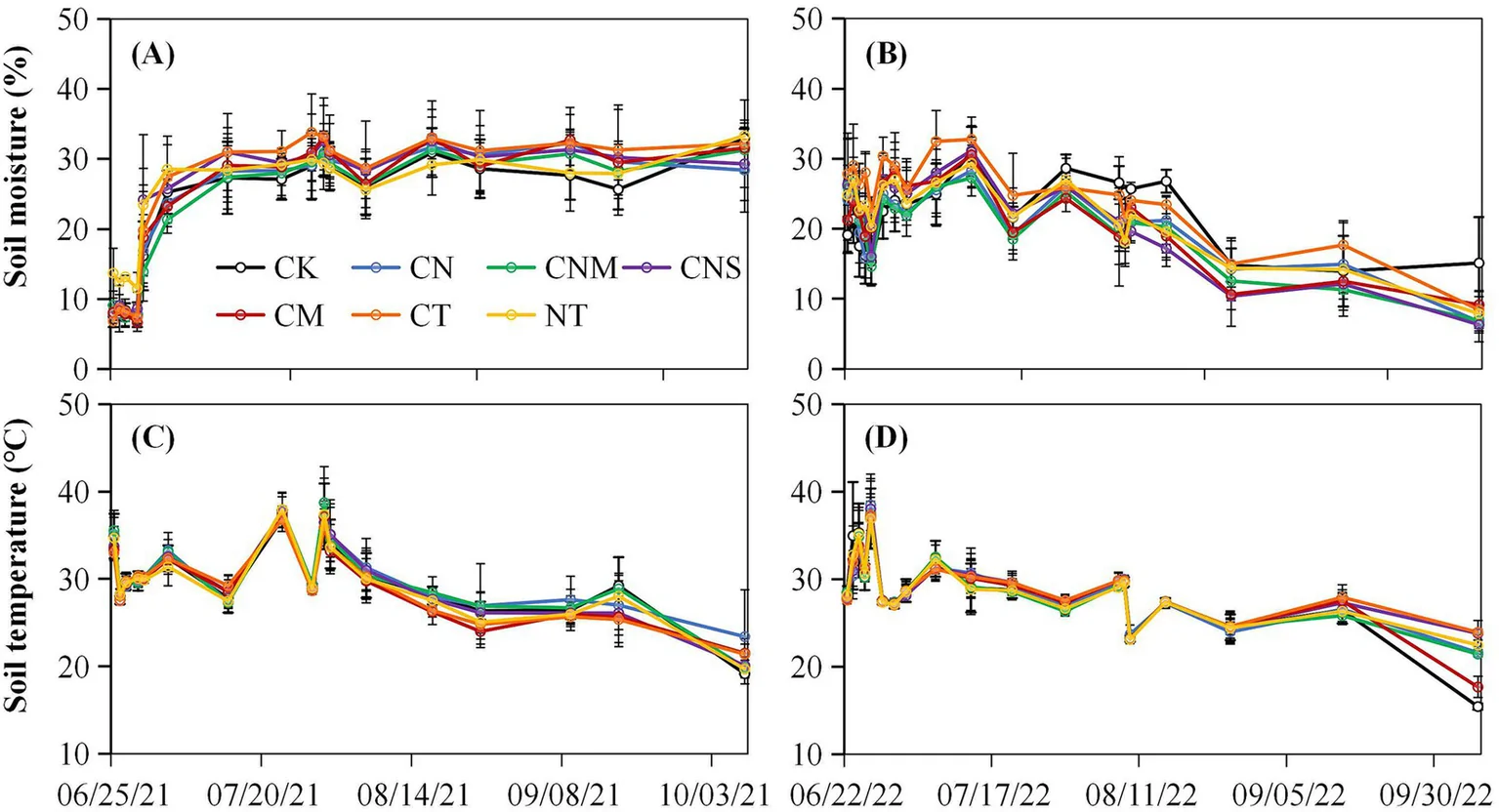
Soil moisture in 2021 **(A)** and 2022 **(B)** and soil temperature in 2021 **(C)** and 2022 **(D)** under different fertilization treatments during the maize growing seasons. CK, no N fertilizer; CN, conventional urea; CNM, 75% urea N plus 25% composted poultry manure N; CNS, 75% urea N plus 25% composted slurry N; CM, 100% composted poultry manure N; CT, 75% urea N plus 25% straw N with deep tillage; NT, 75% urea N plus 25% straw N with no tillage. Bars represent standard deviation of the mean (*n* = 3).

Soil NH_4_^+^-N content was significantly affected by fertilization treatment, but not by year or the Treatment × Year interaction (Figure 2A). Averaged across two years, the CN, CNM, CM, CT and NT treatments significantly increased soil NH_4_^+^-N content by 38.9–70.3% relative to CK, whereas CNS did not differ significantly from CK. No significant differences were observed among N fertilization treatments, except that soil NH_4_^+^-N contents under CNM and CM treatments were significantly higher than that under CNS by 28.5 and 34.2%, respectively (Figure 2A). Soil NO_3_^−^-N content significantly varied with fertilization treatment and year, but the Treatment × Year interaction was not significant (Figure 2B). Averaged across treatments, soil NO_3_^−^-N content was 17.0% higher in 2022 than in 2021. Averaged across two years, all N fertilization treatments except CNS significantly increased soil NO_3_^−^-N content by 31.0–47.7% compared to CK. Soil NO_3_^−^-N contents under CN and CT treatments were 25.0 and 23.3% higher than that under CNS, respectively, while the other N fertilization treatments did not significantly differ from each other (Figure 2B).

**Figure 2:**
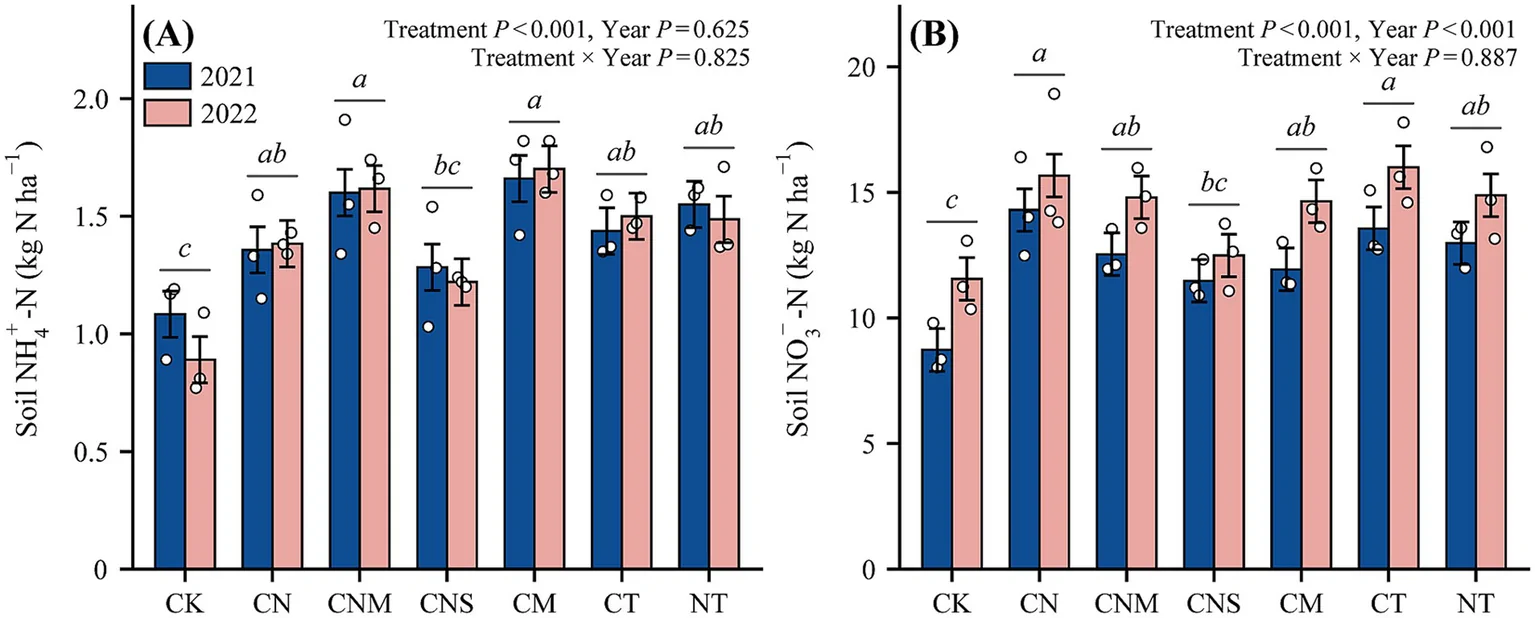
Soil NH_4_^+^-N **(A)** and NO_3_^−^-N **(B)** contents under different fertilization treatments during the maize growing seasons in 2021 and 2022. CK, no N fertilizer; CN, conventional urea; CNM, 75% urea N plus 25% composted poultry manure N; CNS, 75% urea N plus 25% composted slurry N; CM, 100% composted poultry manure N; CT, 75% urea N plus 25% straw N with deep tillage; NT, 75% urea N plus 25% straw N with no tillage. Different lowercase letters indicate Tukey-adjusted differences among treatment marginal means averaged across 2 years at the level of 0.05 based on linear mixed-effects model. Bars represent model-based standard errors of marginal means (*n* = 3).

### 3.2 GHG emissions

The N_2_O fluxes under N fertilization treatments during the maize growing season followed a similar seasonal pattern in both years and peaked mainly after each fertilization event and when soil moisture was high (Figures 1A,B, 3A,B). The peak N_2_O fluxes from CK, CN, CNM, CNS, CM, CT and NT treatments lied between 12.2–14.1, 28.1–59.6, 24.8–31.6, 25.4–31.1, 20.6–32.0, 18.8–66.0 and 23.7–49.0 g N ha^−1^ d^−1^, respectively (Figures 3A,B). In contrast, the CO_2_ fluxes under N fertilization treatments during the maize growing season peaked when soil temperature was high in late June, late July and early August in both years (Figures 1C,D, 3C,D). The peak CO_2_ fluxes from CK, CN, CNM, CNS, CM, CT and NT lied between 32.4–69.5, 46.8–117.5, 65.9–80.5, 50.6–87.9, 65.3–143.2, 80.7–130.9 and 83.4–150.9 kg C ha^−1^ d^−1^, respectively (Figures 3C,D).

**Figure 3:**
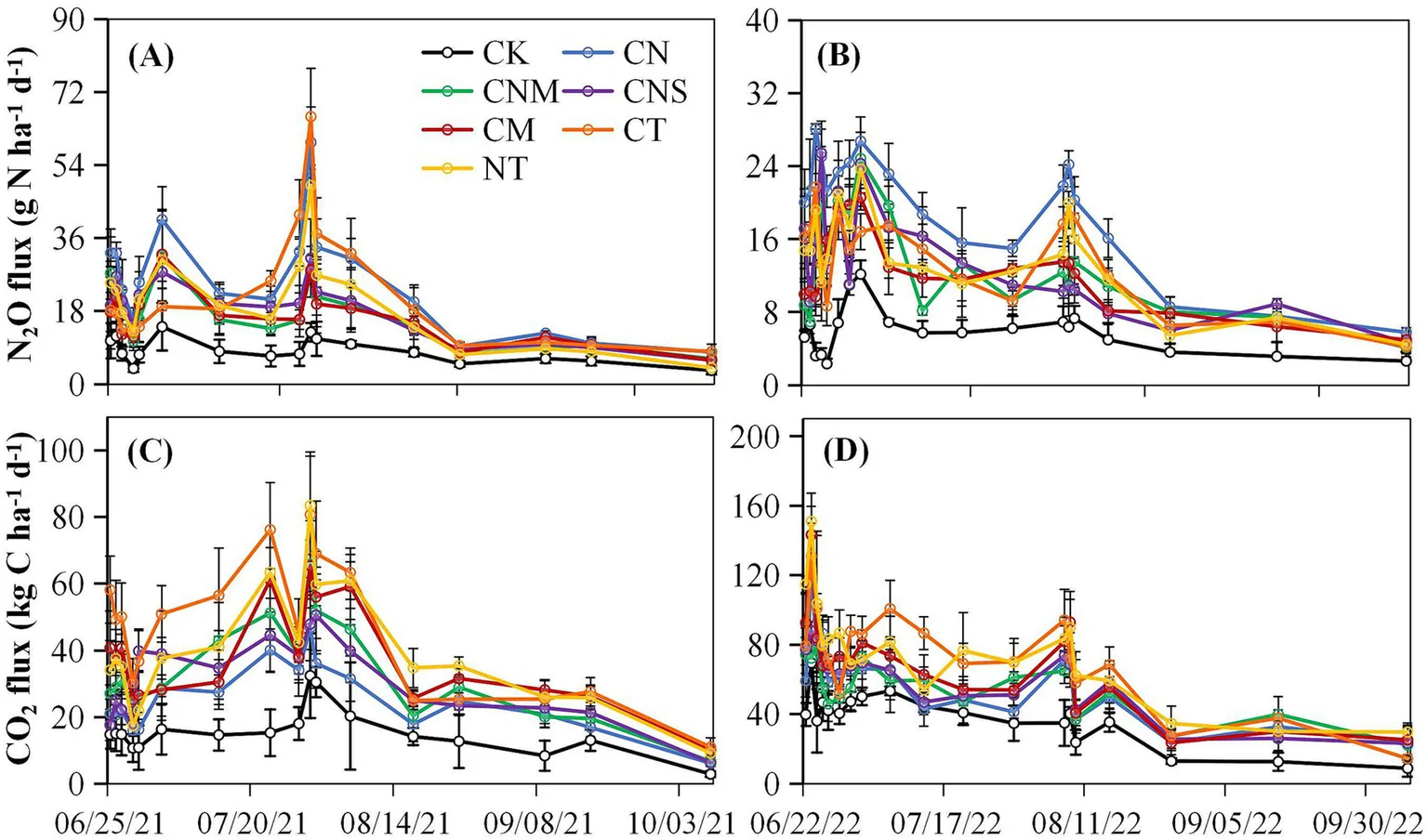
Soil N_2_O fluxes in 2021 **(A)** and 2022 **(B)** and CO_2_ fluxes in 2021 **(C)** and 2022 **(D)** under different fertilization treatments during the maize growing seasons. CK, no N fertilizer; CN, conventional urea; CNM, 75% urea N plus 25% composted poultry manure N; CNS, 75% urea N plus 25% composted slurry N; CM, 100% composted poultry manure N; CT, 75% urea N plus 25% straw N with deep tillage; NT, 75% urea N plus 25% straw N with no tillage. Bars represent standard deviation of the mean (*n* = 3).

Fertilization treatment, year and their interaction significantly affected seasonal cumulative N_2_O emissions (Table 3). Averaged across treatments, cumulative N_2_O emissions were 29.2% lower in 2022 than in 2021. In 2021, N_2_O emissions from CNM, CNS, CM and NT were significantly lower than those from CN by 22.9–30.2%. The CT treatment had significantly greater N_2_O emissions than CNM, CNS and CM by 18.2–27.9%, but did not significantly differ from NT. In 2022, all organic-amended treatments had significantly lower N_2_O emissions than CN by 25.1–29.4%, with no significant differences from one another. In addition, fertilization treatment and year significantly affected both cumulative CO_2_ emissions and GWP, while the Treatment × Year interaction was not significant for either CO_2_ emissions or GWP. Averaged across treatments, cumulative CO_2_ emissions and GWP were 60.3 and 55.3% higher in 2022 than in 2021, respectively. Averaged across two years, CM, CT and NT had significantly greater cumulative CO_2_ emissions by 22.6–44.5% and greater GWP by 19.8–41.1% when compared with CN. Among the organic-amended treatments, CT led to significantly higher CO_2_ emissions and GWP than CNM, CNS and CM treatments by 17.8–32.3% and 17.7–31.5%, respectively, while it did not significantly differ from NT (Table 3).

**Table 3:** Seasonal cumulative N_2_O and CO_2_ emissions and global warming potentials (GWPs) under different fertilization treatments during the maize growing seasons in 2021 and 2022.

Treatment	N_2_O emission (kg N ha^−1^)			CO_2_ emission (t C ha^−1^)			GWP (t CO_2_-eq ha^−1^)		
	2021	2022	Avg.	2021	2022	Avg.	2021	2022	Avg.
CK	0.78 (0.03)*^a^* d*^b^*	0.54 (0.02) c	0.66 (0.03)	1.45 (0.27)	3.01 (0.25)	2.23 (0.21) e	5.66 (1.01)	11.25 (0.90)	8.46 (0.77) e
CN	2.09 (0.18) a	1.53 (0.08) a	1.81 (0.11)	2.48 (0.24)	4.63 (0.32)	3.55 (0.28) d	9.97 (0.88)	17.63 (1.20)	13.80 (1.03) d
CNM	1.46 (0.11) c	1.14 (0.05) b	1.30 (0.08)	3.10 (0.14)	4.93 (0.50)	4.02 (0.29) cd	12.00 (0.57)	18.57 (1.84)	15.29 (1.11) cd
CNS	1.58 (0.08) c	1.15 (0.07) b	1.36 (0.07)	3.01 (0.34)	4.75 (0.39)	3.88 (0.18) cd	11.71 (1.26)	17.90 (1.41)	14.80 (0.68) cd
CM	1.52 (0.08) c	1.08 (0.04) b	1.30 (0.04)	3.52 (0.09)	5.19 (0.43)	4.36 (0.25) bc	13.56 (0.38)	19.51 (1.57)	16.53 (0.93) bc
CT	1.86 (0.14) ab	1.15 (0.11) b	1.51 (0.04)	4.17 (0.57)	6.10 (0.61)	5.13 (0.58) a	16.07 (2.02)	22.85 (2.28)	19.46 (2.11) a
NT	1.61 (0.16) bc	1.14 (0.05) b	1.38 (0.10)	3.82 (0.14)	5.93 (0.54)	4.87 (0.22) ab	14.69 (0.53)	22.22 (1.97)	18.45 (0.83) ab
*F* and *p* values of fixed effects									
Treatment	*F*(6, 12) = 79.19, *p* < 0.001			*F*(6, 12) = 54.31, *p* < 0.001			*F*(6, 12) = 58.23, *p* < 0.001		
Year	*F*(1, 14) = 257.22, *p* < 0.001			*F*(1, 14) = 388.67, *p* < 0.001			*F*(1, 14) = 369.97, *p* < 0.001		
Treatment × Year	*F*(6, 14) = 4.31, *p* = 0.011			*F*(6, 14) = 0.80, *p* = 0.589			*F*(6, 14) = 0.73, *p* = 0.634		

^a^Numbers in brackets are standard deviations (*n* = 3).

^b^Different letters indicate Tukey-adjusted differences among treatments at the level of 0.05 based on linear mixed-effects model. For N_2_O emissions, letters compare treatments within each year due to significant Treatment × Year interaction; for CO_2_ emissions and GWP, letters compare treatment marginal means averaged across two years due to insignificant Treatment × Year interaction.

CK, no N fertilizer; CN, conventional urea; CNM, 75% urea N plus 25% composted poultry manure N; CNS, 75% urea N plus 25% composted slurry N; CM, 100% composted poultry manure N; CT, 75% urea N plus 25% straw N with deep tillage; NT, 75% urea N plus 25% straw N with no tillage.

### 3.3 SOC stock

Fertilization treatment exerted significant effects on SOC stock, whereas the effects of year and the Treatment × Year interaction were not significant (Figure 4). Averaged across two years, all organic-amended treatments significantly increased SOC stock by 16.7–46.2% relative to CN. Specifically, CM treatment had the highest marginal mean SOC stock and was significantly higher than CNM, CT and NT by 25.2%, 17.5 and 15.9%, respectively. Besides, SOC stock under CNS was significantly greater than that under CNM by 17.1%. No significant differences were observed among CNM, CT and NT or among CNS, CT and NT (Figure 4).

**Figure 4:**
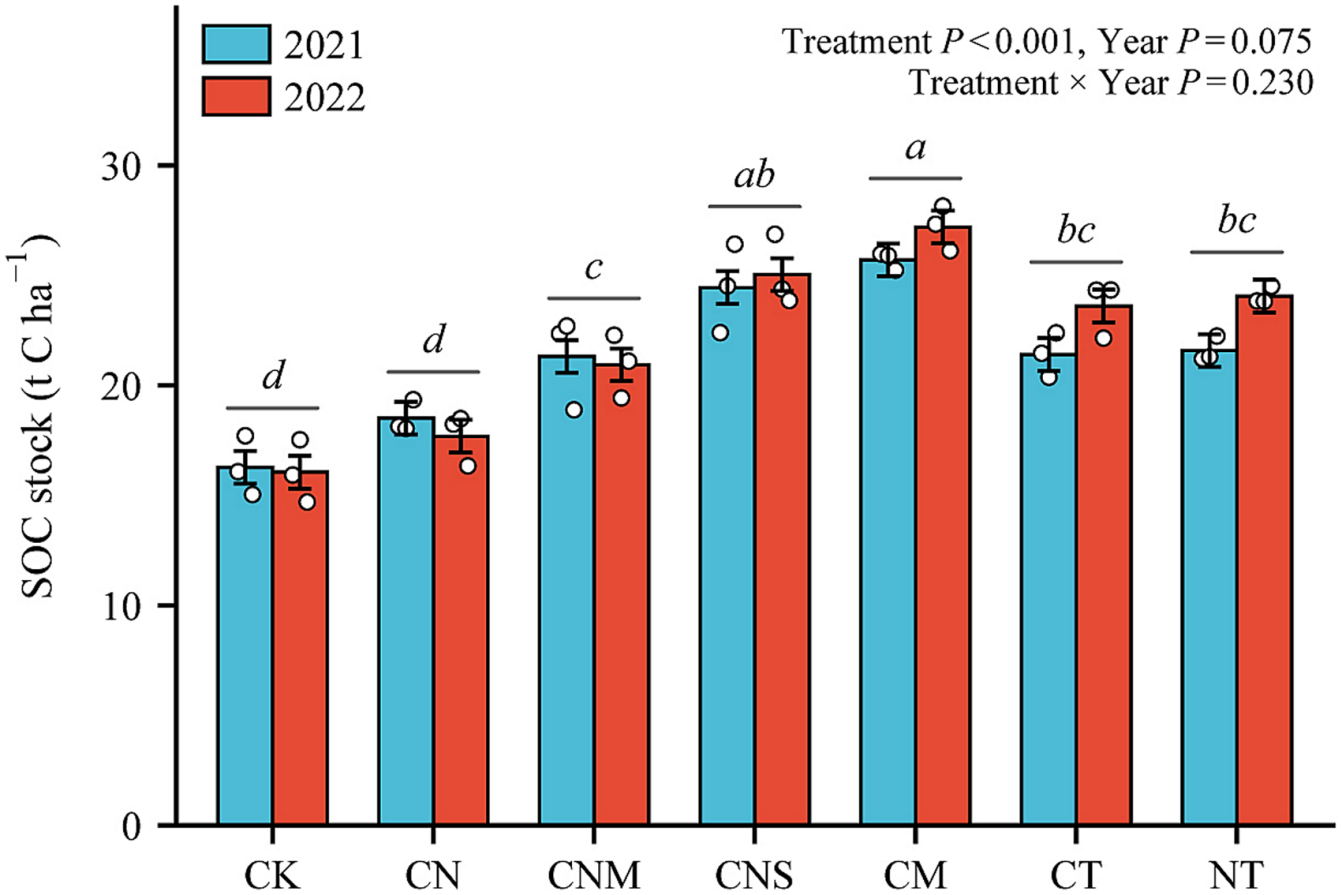
Soil organic carbon (SOC) stocks in 2021 **(A)** and 2022 **(B)**. CK, no N fertilizer; CN, conventional urea; CNM, 75% urea N plus 25% composted poultry manure N; CNS, 75% urea N plus 25% composted slurry N; CM, 100% composted poultry manure N; CT, 75% urea N plus 25% straw N with deep tillage; NT, 75% urea N plus 25% straw N with no tillage. Different lowercase letters indicate Tukey-adjusted differences among treatment marginal means averaged across 2 years at the level of 0.05 based on linear mixed-effects model. Bars represent model-based standard errors of marginal means (*n* = 3).

Fertilization treatment significantly affected SOC sequestration rate, SOC sequestration efficiency and net GWP, whereas neither the year effect nor the Treatment × Year interaction was significant for these variables (Table 4). Averaged across two years, all organic-amended treatments had significantly higher SOC sequestration rates than CN by 155.6–424.7%. Among organic-amended treatments, CM had the highest SOC sequestration rate, which was significantly greater than CNM, CT and NT by 55.1–105.3%. The SOC sequestration rate under CNS was 71.0% higher than that under CNM. No significant differences were observed in the SOC sequestration rates among CNM, CT and NT treatments. Similarly, averaged across two years, SOC sequestration efficiency under CNM, CNS, CM and NT treatments were significantly higher than that under CN by 87.0–200.0%, while CT did not significantly differ from CN. The SOC sequestration efficiency reached the highest level under CM, which significantly exceeded the other organic-amended treatments by 38.6–69.8%. Notably, net GWP was positive under CK and CN treatments, but shifted to negative under organic-amended treatments in both years. Averaged across two years, significant reductions in net GWP were observed only under CNS and CM treatments relative to CN. Among the organic-amended treatments, net GWP was lowest under CM and highest under CT. Specifically, CM was significantly lower than CT, but did not significantly differ from CNM, CNS or NT (Table 4).

**Table 4:** Soil organic carbon (SOC) sequestration rate, seasonal SOC sequestration efficiency and seasonal net global warming potential (GWP) under different fertilization treatments during the maize growing seasons in 2021 and 2022.

Treatment	SOC sequestration rate (t ha^−1^ yr.^−1^)			SOC sequestration efficiency (%)			Net GWP (t CO_2_-eq ha^−1^)		
	2021	2022	Avg.	2021	2022	Avg.	2021	2022	Avg.
CK	-	-	-	-	-	-	12.10 (5.22)	8.01 (3.44)	10.05 (3.29) a
CN	0.16 (0.05)*^a^*	0.11 (0.08)	0.13 (0.01) d*^b^*	6.0 (0.8)	5.5 (2.7)	5.8 (0.9) c	4.24 (4.63)	1.58 (3.07)	2.91 (3.77) ab
CNM	0.36 (0.15)	0.33 (0.10)	0.34 (0.08) c	13.9 (4.2)	9.3 (3.2)	11.6 (2.7) b	−4.72 (3.86)	−5.48 (2.89)	−5.10 (0.69) bcd
CNS	0.58 (0.14)	0.60 (0.11)	0.59 (0.03) ab	12.4 (3.2)	12.7 (2.0)	12.5 (0.8) b	−6.76 (6.13)	−7.78 (5.84)	−7.27 (0.74) cd
CM	0.67 (0.03)	0.74 (0.07)	0.71 (0.05) a	16.2 (0.6)	18.5 (1.7)	17.4 (1.1) a	−11.14 (3.38)	−12.36 (2.55)	−11.75 (1.56) d
CT	0.37 (0.07)	0.50 (0.08)	0.44 (0.08) bc	9.8 (1.9)	10.6 (1.7)	10.2 (1.8) bc	−1.51 (4.57)	−3.26 (4.21)	−2.39 (3.48) bc
NT	0.38 (0.04)	0.53 (0.03)	0.46 (0.03) bc	10.2 (1.6)	11.4 (0.4)	10.8 (1.0) b	−3.01 (1.91)	−4.34 (3.06)	−3.68 (1.68) bcd
*F* and *p* values of fixed effects									
Treatment	*F*(5, 10) = 30.98, *p* < 0.001			*F*(5, 10) = 16.00, *p* < 0.001			*F*(6, 12) = 18.10, *p* < 0.001		
Year	*F*(1, 12) = 2.82, *p* = 0.119			*F*(1, 12) = 0.01, *p* = 0.909			*F*(1, 14) = 2.10, *p* = 0.169		
Treatment × Year	*F*(5, 12) = 1.43, *p* = 0.282			*F*(5, 12) = 1.62, *p* = 0.227			*F*(6, 14) = 0.12, *p* = 0.992		

^a^Numbers in brackets are standard deviations (*n* = 3).

^b^Different letters indicate Tukey-adjusted differences among treatment marginal means averaged across two years due to insignificant Treatment × Year interaction at the level of 0.05 based on linear mixed-effects model.

CK, no N fertilizer; CN, conventional urea; CNM, 75% urea N plus 25% composted poultry manure N; CNS, 75% urea N plus 25% composted slurry N; CM, 100% composted poultry manure N; CT, 75% urea N plus 25% straw N with deep tillage; NT, 75% urea N plus 25% straw N with no tillage.

### 3.4 Soil microbial communities

The organic-amended treatments generally showed a greater abundance of functional genes involved in soil C and N cycling than the CN treatment (Figure 5). Compared with CN, the total abundance of C degradation genes under the CNM, CNS, CM, CT and NT treatments was 49.4%, 23.8%, 82.5%, 57.7 and 26.0% higher, respectively, without significant differences. The abundance of hemicellulose degradation genes (sum of *abfA*, *manB* and *xylA*) increased significantly under CM by 118%, driven mainly by *abfA*, which was also significantly higher under CNM, CM and CT than under CN by 138%, 167 and 208% (Figure 6A). The total abundance of C fixation genes under the CNM, CNS, CM, CT and NT treatments was 34.4%, 38.7%, 53.0%, 25.3 and 20.6% higher than under CN, respectively, with no significant differences. Among individual C fixation genes, only *acsA* under CM significantly increased by 69% (Figure 6C). The total abundance of N cycling genes under the CNM, CNS, CM, CT and NT treatments was 60.4%, 2.7%, 76.7%, 49.0 and 37.1% higher than under CN, respectively, with no significant differences. Specifically, CM significantly increased the abundance of *napA*, *narG* and *ureC* by 152%, 70 and 102%, respectively. Besides, *nirS2* was significantly higher under CNM and CT by 143 and 167%, respectively, and *nirK1* was significantly higher under CT by 293%. No significant differences were observed in the abundance of *amoA/B* (sum of *amoA1*, *amoA2* and *amoB*) as well as *nosZ* (sum of *nosZ1* and *nosZ2*) between organic-amended treatments and CN. However, it should be noted that, in comparison with CN, the CNM, CNS, CM, CT and NT treatments had 32.6%, 57.4%, 51.7%, 45.4 and 40.6% lower abundance of *amoA2*, and 81%, 27%, 99%, 66 and 30% higher abundance of *nosZ*, respectively (Figure 6B).

**Figure 5:**
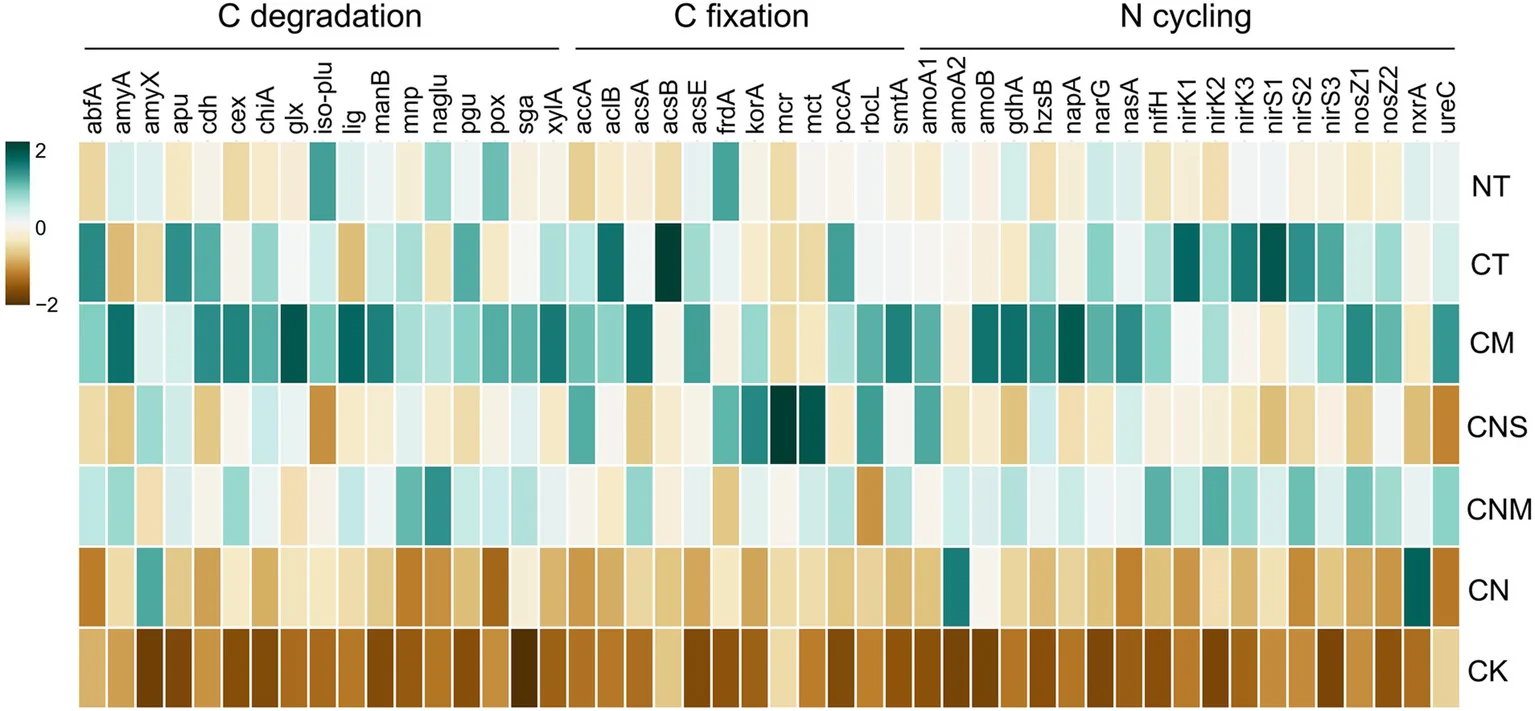
Absolute abundance heatmap of functional genes involved in C degradation, C fixation and N cycling in soil under different treatments. CK, no N fertilizer; CN, conventional urea; CNM, 75% urea N plus 25% composted poultry manure N; CNS, 75% urea N plus 25% composted slurry N; CM, 100% composted poultry manure N; CT, 75% urea N plus 25% straw N with deep tillage; NT, 75% urea N plus 25% straw N with no tillage.

**Figure 6:**
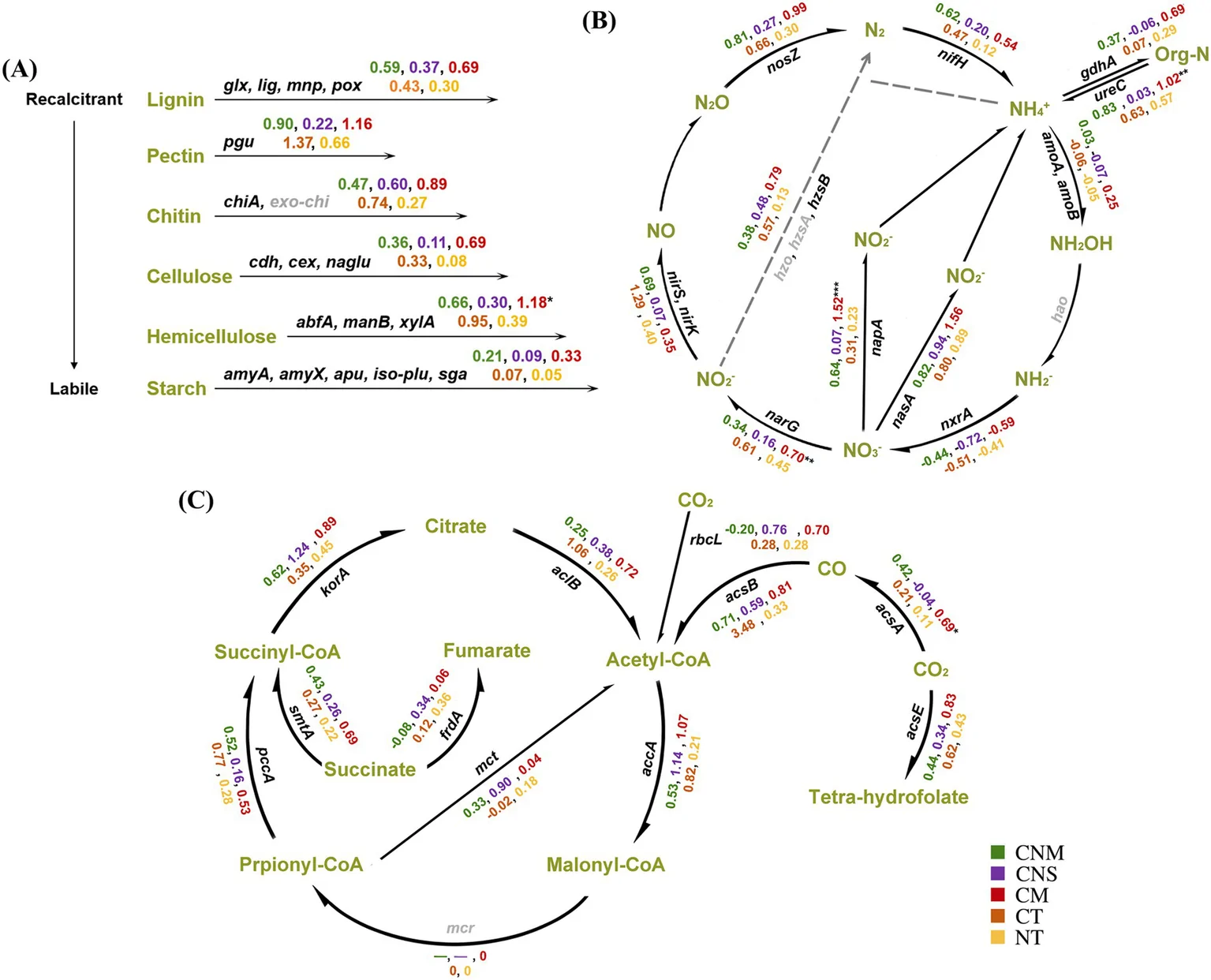
Percentage change in the abundance of functional genes involved in C degradation **(A)**, N cycling **(B)** and C fixation **(C)** in soil under organic-amended treatments (i.e., CNM, CNS, CM, CT and NT) relative to CN. CN, conventional urea; CNM, 75% urea N plus 25% composted poultry manure N; CNS, 75% urea N plus 25% composted slurry N; CM, 100% composted poultry manure N; CT, 75% urea N plus 25% straw N with deep tillage; NT, 75% urea N plus 25% straw N with no tillage. *Significant differences between CN and organic-amended treatments at *p* ≤ 0.05; **significant differences between CN and organic-amended treatments at *p* ≤ 0.01; ***significant differences between CN and organic-amended treatments at *p* ≤ 0.001.

The NMDS analysis (Stress = 0.068, Adonis *p* = 0.004) indicated that the soil functional microbial communities under organic-amended treatments were significantly separated from CN along the NMDS1 axe. While the functional microbial communities of CNM, CNS, CT and NT partially overlapped with CN, significant separation was observed between CM and CN along the NMDS2 axe (Figure 7A). The results of RDA showed that the assemblage of soil functional microbial communities across different fertilization treatments was mainly regulated by soil organic matter (SOM, 20.1%), TN (15.1%) and soil NO_3_^−^–N (4.3%) (Figure 7B). Similarly, Mantel test demonstrated that SOM, TN and NO_3_^−^-N were the major drivers of C degradation, C fixation and denitrification communities, whereas the nitrification community was significantly affected by TN and NO_3_^−^-N and other microbial communities involved in N cycling by soil temperature. Besides, significantly positive correlations were observed between TN and SOM, TN and soil NH_4_^+^–N, and SOM and soil EC (Figure 7C).

**Figure 7:**
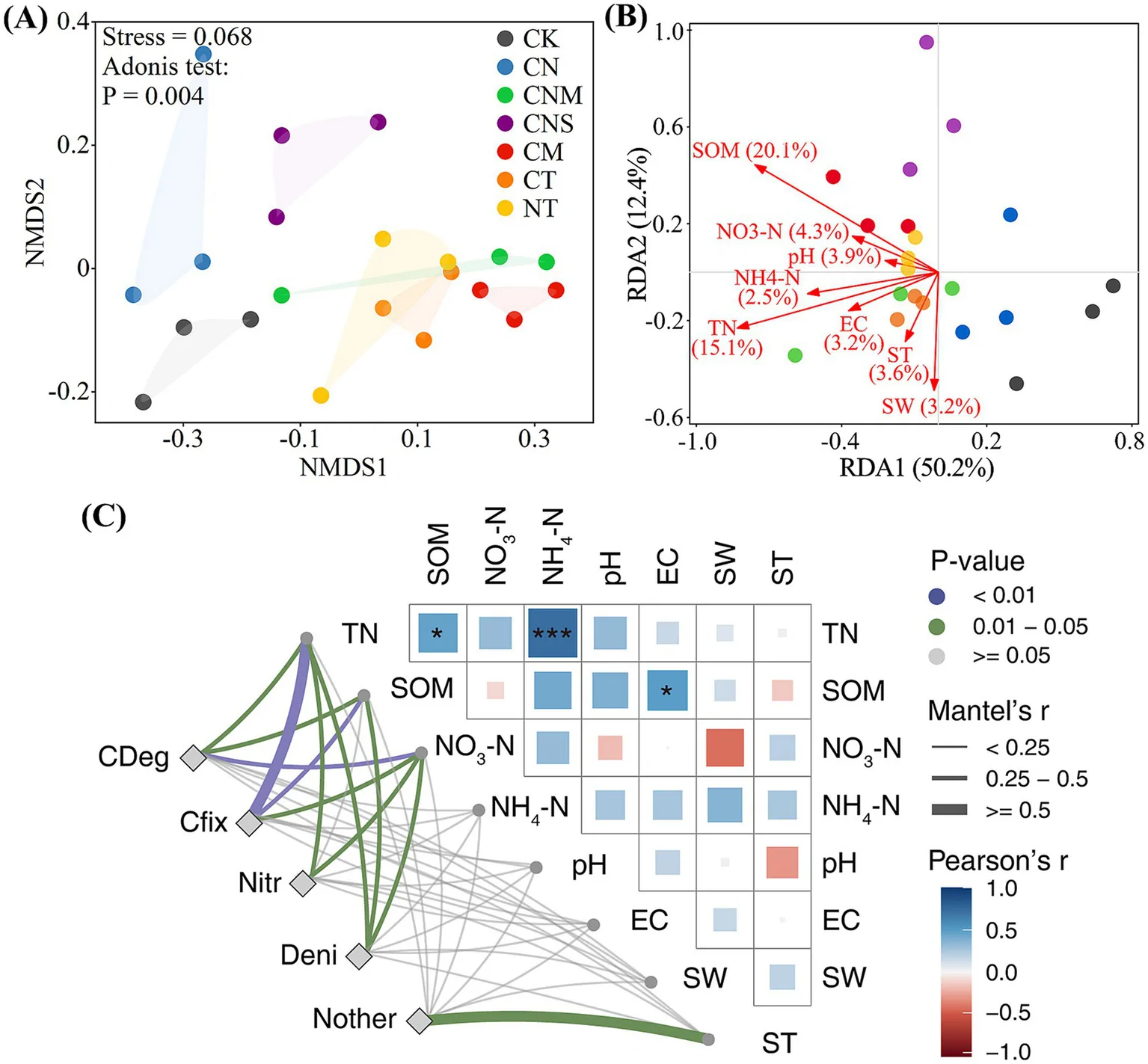
Soil community assemblage based on NMDS analysis **(A)**, and correlations between soil microbial community assemblage and soil properties shown by db-RDA **(B)** and Mantel test **(C)**. Values in parentheses represent explained variation of each environmental factor in microbial community composition. SOM, soil organic matter; TN, soil total N; NO3-N, soil nitrate-N; NH4-N, soil ammonium-N; EC, soil electrical conductivity; pH, soil pH; SW, soil water content; ST, soil temperature; CDeg, C degradation communities; Cfix, C fixation communities; Nitr, nitrifiers harboring functional genes including *amoA1*, *amoA2*, *amoB*, *hao* and *nxrA*; Deni, denitrifiers harboring functional genes including *narG*, *nirK1*, *nirK2*, *nirK3*, *nirS1*, *nirS2*, *nirS3*, *nosZ1* and *nosZ2*; Nother, other microbial communities involved in N cycling. CK, no N fertilizer; CN, conventional urea; CNM, 75% urea N plus 25% composted poultry manure N; CNS, 75% urea N plus 25% composted slurry N; CM, 100% composted poultry manure N; CT, 75% urea N plus 25% straw N with deep tillage; NT, 75% urea N plus 25% straw N with no tillage. *p ≤ 0.05; **p ≤ 0.01; ***p ≤ 0.001.

### 3.5 PLS-PM analysis

The results of PLS-PM analysis indicated that N fertilization was significantly positively associated with soil TN, soil available N (i.e., NO_3_^−^-N and NH_4_^+^–N) and SOC (Figure 8). Soil TN was significantly positively correlated with all functional gene profiles. Soil available N was significantly positively associated with N_2_O emissions, while soil climate (i.e., soil temperature and water content) was significantly negatively associated with both N_2_O and CO_2_ emissions. The C fixation and C degradation gene profiles were significantly positively correlated with SOC and CO_2_ emissions, respectively, whereas N cycling gene profile was significantly negatively correlated with N_2_O emissions. Besides, SOC was significantly positively associated with CO_2_ emissions (Figure 8).

**Figure 8:**
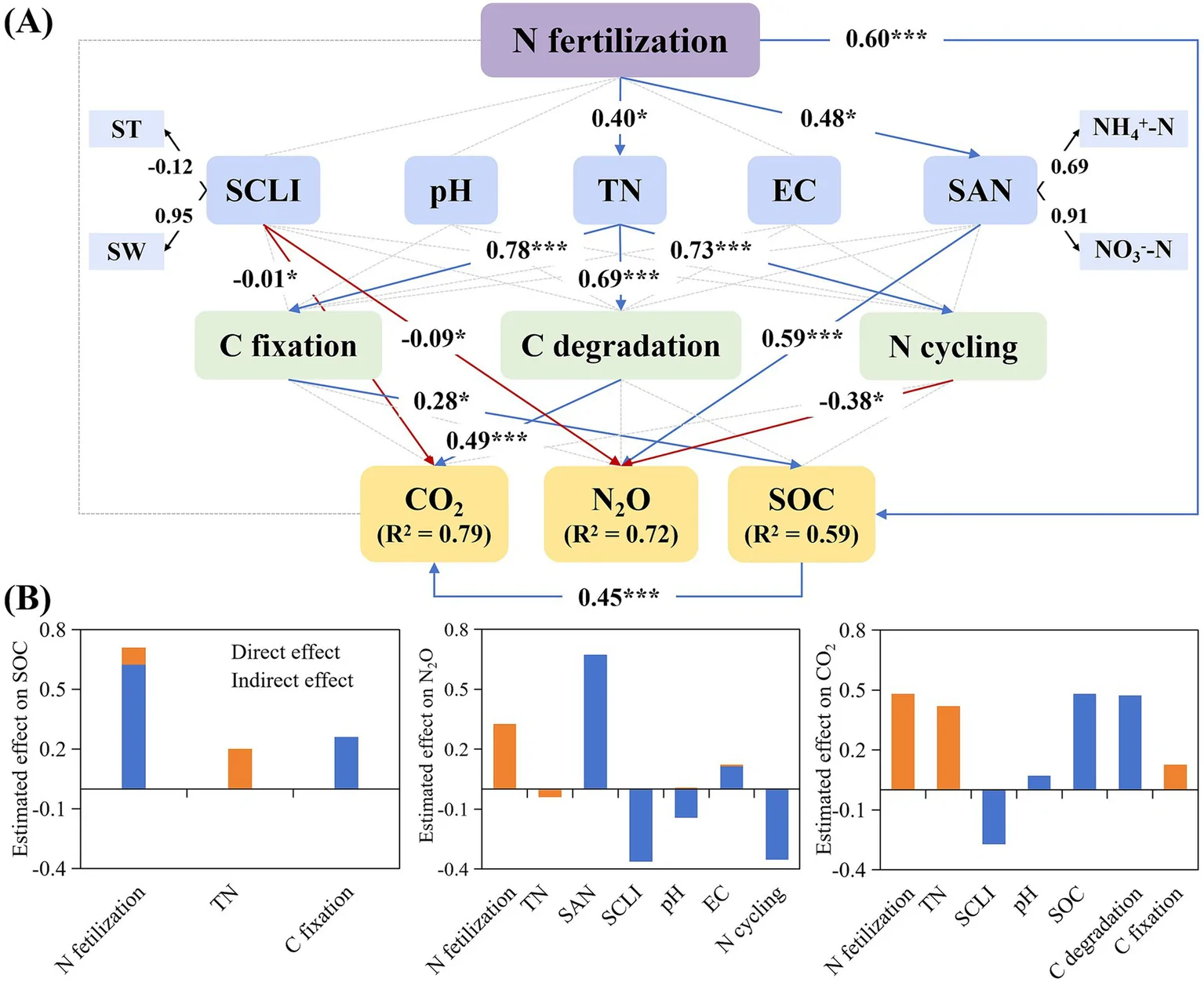
Partial least squares-path modeling (PLS-PM) analysis showing the effects of different factors including N fertilization, soil properties and soil functional microbial communities on seasonal cumulative greenhouse gas (i.e., N_2_O and CO_2_) emissions and SOC sequestration. **(A)** Directed graph of PLS-PM. Each box represent an observed variable or a latent variable. Blue and red lines denote positive and negative effects, respectively. Dashed gray lines indicate no significant effect (*p* > 0.05). Significant path coefficients were indicated by *p ≤ 0.05, **p ≤ 0.01, ***p ≤ 0.001. The goodness of fit (GoF) value of the model was 0.59. **(B)** Direct and indirect effects of N fertilization, soil properties and soil functional microbial communities on SOC, N_2_O and CO_2_. TN, soil total N; SAN, soil available N; NO_3_^−^-N, soil nitrate-N; NH_4_^+^-N, soil ammonium-N; SCLI, soil climate; ST, soil temperature; SW, soil water content; EC, soil electrical conductivity; pH, soil pH. C fixation, C fixation communities; C degradation, C degradation communities; N cycling, N cycling communities.

## 4 Discussion

### 4.1 Effects of organic amendments on soil climate and nutrient availability

The greater soil moisture in 2021 than 2022 was mainly due to the higher seasonal precipitation in 2021 (74.4 mm) compared with 2022 (33.7 mm) (Supplementary Figure 1). Consistent with previous research, organic-amended treatments generally had higher soil moisture than CN treatment in this study, probably attributed to improved soil porosity and water holding capacity as well as decreased soil water evaporation under manure application and straw return (Cui et al., 2014; Wang et al., 2024).

Compared with chemical N fertilization, organic amendment was observed to stimulate N immobilization via enhanced C availability and microbial activity in soil (Qiu et al., 2016). In addition, the increased soil moisture and microbial oxygen consumption induced by organic matter inputs could further inhibit nitrification in soil, thereby leading to the significantly reduced soil NO_3_^−^-N contents under CNS and CM treatments than CN in this study. Wang et al. (2019) reported that organic manure stimulated N immobilization in soil more than straw attributed to the increase in both SOM and microbial assimilation, which could be the cause of the significantly lower soil NO_3_^−^-N content under CNS than CT in this study. On the other hand, it is widely known that the more rapid and greater release of N from chemical fertilizers often leads to higher NH_4_^+^-N loss via NH_3_ volatilization, while organic amendments could result in greater NH_4_^+^-N fixation by clay minerals or lignin-derived phenolic compounds as well as improved plant uptake of NH_4_^+^-N (Cao et al., 2020). This was likely the main reason for the lack of significant differences in soil NH_4_^+^-N contents between CN and organic-amended treatments in this study.

### 4.2 Effects of organic amendments on GHG emissions and SOC stocks

In this study, the N_2_O fluxes were primarily driven by N fertilization and soil moisture, whereas the CO_2_ fluxes were largely controlled by soil temperature, a pattern in alignment with previous studies and possibly due to increased C and N availability and improved microbial activity in soil (Lai et al., 2017; Fan et al., 2022). The organic-amended treatments had significantly lower cumulative N_2_O emissions than CN in this study. Consistently, both field experiments and meta-analysis revealed that organic amendments can reduce the N_2_O emissions from maize systems compared with conventional chemical N fertilization (Zhou et al., 2017; Lv et al., 2020; Ren et al., 2022). The decreased soil N availability, as supported by the lower soil NO_3_^−^-N contents under CNS and CM treatments than CN in this study, and the stimulated microbial N immobilization could result in less available substrates for nitrification or denitrification and ultimately the reduction in N_2_O emissions under organic amendments (Hou et al., 2015; Qiu et al., 2016). In contrast, some other studies reported contradictory findings. Afreh et al. (2018) observed an increase in N_2_O emissions since manure provided readily available C source for denitrifiers to produce more N_2_O. Xia et al. (2018) suggested that N_2_O emissions from upland fields significantly increased following straw return, mainly owing to the activated nitrification and denitrification by additional N substrate derived from straw degradation as well as the promoted denitrification caused by decreased oxygen availability during straw decomposition. These inconsistent conclusions likely resulted from the complex interplays among environmental conditions, management practices and planting systems coupled with different organic matter sources. Budai (2009) reported that the ratio of N_2_O reduced to N_2_ was positively correlated with soil pH, and therefore the alleviated soil acidity under manure application would decrease N_2_O production via improved N_2_O reduction. The findings by Zhou et al. (2017) indicated that straw return reduced N_2_O emissions during the summer-maize season with relatively high precipitation, as both high soil moisture and additionally consumed oxygen by rotting straw resulted in severe soil anoxic condition and N_2_ became the dominant product of soil denitrification. Jiang et al. (2016) argued that the promoted plant uptake of available N in soil may also lead to lower N_2_O emissions. Additionally, previous studies demonstrated that the amendment of organic matter with relatively high C/N ratio or at an optimized N application rate would reduce N_2_O emissions by enhancing soil N immobilization (Huang et al., 2004; Wei et al., 2024). This highlights the importance of optimizing organic amendment practices targeting site-specific conditions to improve soil quality without triggering N_2_O production.

Soil CO_2_ emissions from croplands primarily originate from soil microbial respiration (Chanu et al., 2024). Consistent with previous studies (Lv et al., 2022; Li et al., 2024), organic-amended treatments had higher CO_2_ emissions than CN in this study. Manure application and straw return increase soil CO_2_ emissions by providing more substrates for microbial metabolism directly via elevated SOC content, as supported by the results in this study, and by causing an increase in SOC mineralization via priming effect (Thangarajan et al., 2013). Higher CO_2_ emissions were found under straw return treatments than manure application ones in this study, as a result of greater organic C inputs into soil. In addition, conventional tillage can accelerate SOC mineralization by enhancing microbial respiration through physical disturbance and soil aggregate disruption (Peterson et al., 2019), consequently leading to the relatively higher CO_2_ emissions under CT than NT in this study. As CO_2_ remained the dominant contributor to GWP across different treatments, a consistent pattern was found between GWPs and CO_2_ dynamics.

Compared to CN, organic-amended treatments, especially manure application, substantially increased SOC stocks as expected in this study. The lowest SOC stocks were observed under CNM among different organic-amended treatments, mainly because CNM had significantly lower exogenous organic C inputs into soil. Similar with previous findings by Zhou et al. (2016), the SOC stocks under straw return treatments were significantly lower than those under CM in this study, despite higher organic C inputs from the former. The elevated CO_2_ emissions observed under straw return relative to manure application implied that the enhanced C output partially offset the additional C inputs from straw (Liu et al., 2014). Compared with fresh straw, which contains large amounts of relatively labile polysaccharides (cellulose and hemicellulose) and decomposes rapidly, composted manure has undergone prior biodegradation and retains a greater proportion of humified, aromatic and phenolic organic compounds, and therefore decomposes more slowly (Zhu et al., 2021). Because soil microorganisms preferentially utilize labile C for rapid growth (Di Lonardo et al., 2017; Cui et al., 2020), straw-derived labile C stimulates soil microbial mineralization to a greater extent, whereas C in composted manure is mineralized more slowly. This difference in decomposability may partly explain the higher SOC sequestration rates and efficiency under the CM and CNS treatments than in the straw return treatments in this study. In addition, the SOC sequestration rates and efficiency both improved under organic-amended treatments relative to CN, and the positive net GWP under CN became negative under organic amendments, suggesting that the CO_2_-equivalent SOC gains exceeded the warming contribution of N_2_O emissions and reduced the field-scale net climate impact. Although the SOC sequestration rates and efficiency were comparable between straw return and CNM treatments, the former exhibited higher net GWP in both years primarily due to their elevated N_2_O emissions.

Consistent with previous research, this study demonstrated that both manure application and straw return can reduce chemical fertilizer inputs while maintaining maize yield compared to conventional N fertilization (Supplementary Table 2), owing to the enhanced SOM, reduced bulk density and improved nutrient retention under organic amendments (Lv et al., 2019; Wang X. et al., 2020). When taking maize yield, SOC sequestration and net GWP all into consideration, CM treatment demonstrated the most superior comprehensive performance in both enhancing soil fertility and mitigating environmental risks without yield loss.

### 4.3 Impacts of organic amendments on soil functional microbial communities

In accordance with the results of this study, previous research reported an increase in microbial biomass along with the abundance of functional genes involved in C and N cycling in soil under organic amendments, primarily as a result of stimulated microbial growth and functional gene expression due to improved soil physicochemical properties and increased substrate supply for microbes (Ma et al., 2020; Tang et al., 2023; Andrews et al., 2024). Organic amendments tended to enhance the abundance of functional genes regulating both labile and recalcitrant C degradation in this study. This suggests a potentially heightened microbial capacity for decomposing a wide range of C substrates and a strong potential for simultaneously increased CO_2_ fluxes and SOC (Liu et al., 2020), which is consistent with the results of this study. Among organic-amended treatments, CM and CT exhibited the highest abundance of C degradation genes. This may explain the higher CO_2_ emissions under CM and CT, supported by the significantly positive correlations between C degradation gene profile and CO_2_ emissions revealed by PLS-PM analysis in this study as well as previous study demonstrating a direct correlation between soil CO_2_ emissions and functional gene abundance (Chen and Sinsabaugh, 2021). In this study, manure application treatments tended to demonstrated greater abundance of C fixation genes in soil than straw return. This could be one of the primary reasons for the higher SOC stocks under CM and CNS than CT and NT as C fixation communities play an important role in driving C assimilation and promoting soil C pools (Wen et al., 2024), consistent with the significantly positive associations of C fixation gene profile with SOC by PLS-PM analysis. Moreover, CM exhibited the highest abundance of soil C fixation genes and a greater SOC stock than CNS and CNM. The greater abundance of C fixation genes under CM may have offset its higher CO_2_ emissions and thereby contributed to the greater SOC sequestration.

In consistency with the results of this study, Yang et al. (2024) found a greater positive response of N cycling gene abundance to manure addition than straw return, as manure provided a more balanced supply of readily available C and N and more effectively accelerated the growth of N cycling microbes in soil. While previous studies observed an increase in ammonia-oxidizing bacteria (AOB) abundance with organic amendments (Ouyang et al., 2018; Yang et al., 2024), this study found that *amoA2* abundance tended to be lower under organic-amended treatments than under CN, while *amoA2* abundance was significantly positively correlated with soil N_2_O emissions (Supplementary Figure 2). Given that AOB dominated N_2_O generation in N-rich soils (Di et al., 2009), this tendency suggested reduced nitrification potential, which may have contributed to the mitigated N_2_O emissions under organic amendments. Bi et al. (2023) and Hei et al. (2023) reported a similar decrease in AOB abundance and concurrent reduction in N_2_O emissions under long-term organic manure application, owing to reduced soil available N resulted from slower N release from organic manure and consequently limited AOB growth. Furthermore, *nosZ* gene abundance tended to be higher under organic amendments relative to CN, likely driven by more oxygen-limited conditions associated with enhanced soil moisture and microbial respiration, indicated a greater potential for N_2_O reduction to N_2_, which may have further contributed to the lower N_2_O emissions (Juhanson et al., 2017; Xie et al., 2021).

### 4.4 Microbial mechanisms regulating SOC sequestration and N_2_O mitigation by organic amendments

In line with previous findings (Wang N. et al., 2020; Krause et al., 2025), NMDS analysis in this study showed that the composition of soil functional microbial communities significantly differed between the organic-amendment treatments and CN, which may in turn resulted in distinctive responses of SOC sequestration and GHG emissions. The RDA and Mantel tests further indicated that variations in the C degradation, C fixation and denitrification communities were primarily associated with SOM, TN and NO_3_^−^–N. The increased C inputs under organic amendments may have contributed to the enrichment of C fixation genes. Similarly, Wang Y. et al. (2020) reported that continuous organic matter inputs increased the abundance of soil C fixation genes and promoted SOC accumulation. The RDA and Mantel tests in this study also showed that variation in the microbial communities harboring nitrification genes was primarily correlated with TN and NO_3_^−^-N. The tendency toward lower *amoA2* abundance may be related to the lower NO_3_^−^–N following organic amendments relative to CN, as supported by Yuan et al. (2020).

In addition, consistent with the findings of previous research (Han et al., 2016; Wan et al., 2023), the RDA results in this study revealed that enhanced SOC sequestration under organic amendments was mainly associated with exogenous organic matter inputs and C fixation gene abundance, and lower N_2_O emissions were accompanied by decreased soil N availability (i.e., NO_3_^−^–N), lower *amoA2* abundance and higher *nosZ* abundance, suggesting that reduced N_2_O production potential and greater N_2_O reduction potential may both have contributed to the mitigation of N_2_O emissions (Figure 9).

**Figure 9:**
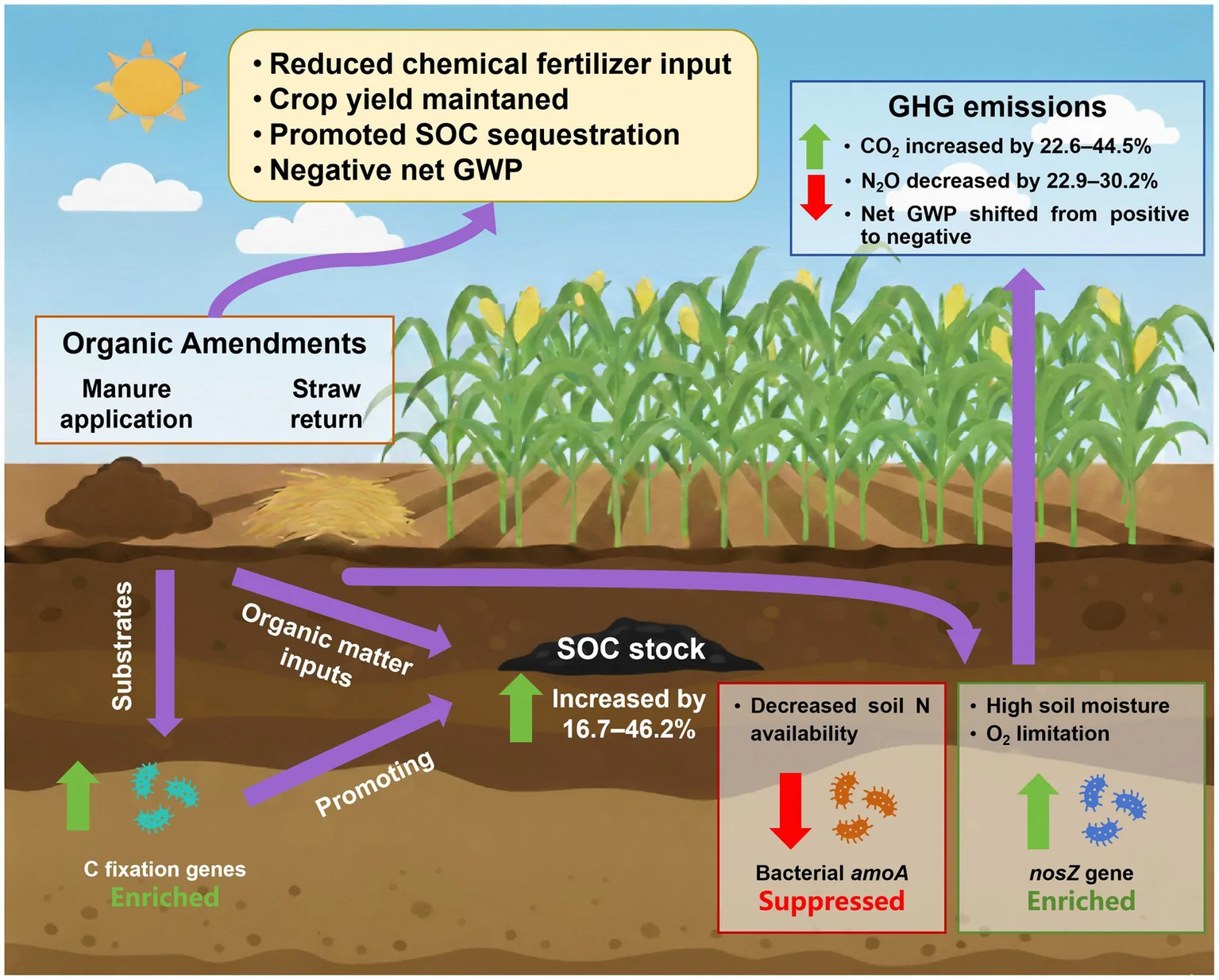
Conceptual diagram illustrating the mechanisms by which organic amendments promoting SOC sequestration and mitigating net global warming potential (GWP) in the maize field.

## 5 Conclusion

Organic amendments (manure application and straw return) shifted the field-scale net GWP from positive to negative during the maize growing season of wheat-maize rotation systems, through enhanced SOC sequestration combined with lower N_2_O emissions. Specifically, manure application was more effective for long-term C sequestration than straw return, consistent with the generally higher C fixation gene abundance in manure-amended soils. Concurrently, decreased soil N availability, and tendencies toward lower bacterial *amoA* abundance and higher *nosZ* abundance under organic amendments suggested that reduced N_2_O production potential and enhanced N_2_O reduction potential may both have contributed to the N_2_O mitigation. In addition, organic amendments maintained crop yield while reducing chemical fertilizer inputs. Therefore, the strategic implementation of organic amendments, particularly long-term manure application, offers a viable pathway toward climate-smart agriculture in wheat-maize rotation systems.

## Data Availability

The original contributions presented in the study are publicly available. This data can be found here: https://doi.org/10.5281/zenodo.23264795.
