# Supplementary material for: Microbial mechanisms underlying the shift of net global warming potential in a maize field under organic amendments
Source: Front Microbiol. 2026 Oct 22;17:1966738. doi: 10.3389/fmicb.2026.1966738 (PMC13736613; doi:10.3389/fmicb.2026.1966738)
Supplement: Supplementary file 1 [file Supplementary_file_1.pdf]

Supplementary Material

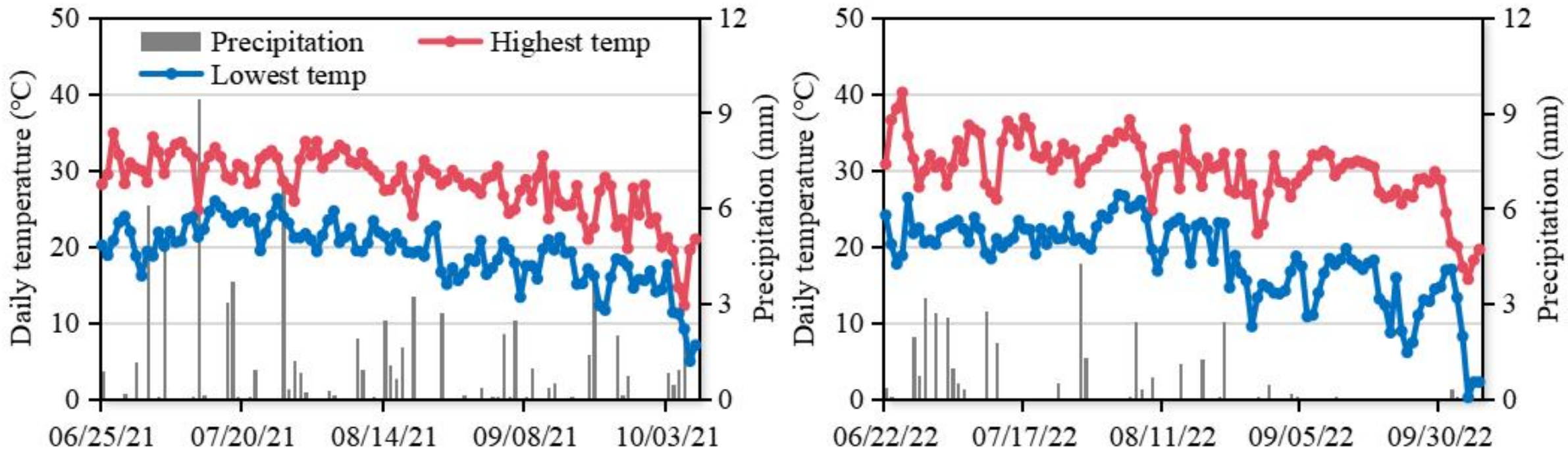

Supplementary Figure 1 Daily air temperature and precipitation during the maize growing seasons in 2021 (**A**) and 2022 (**B**)

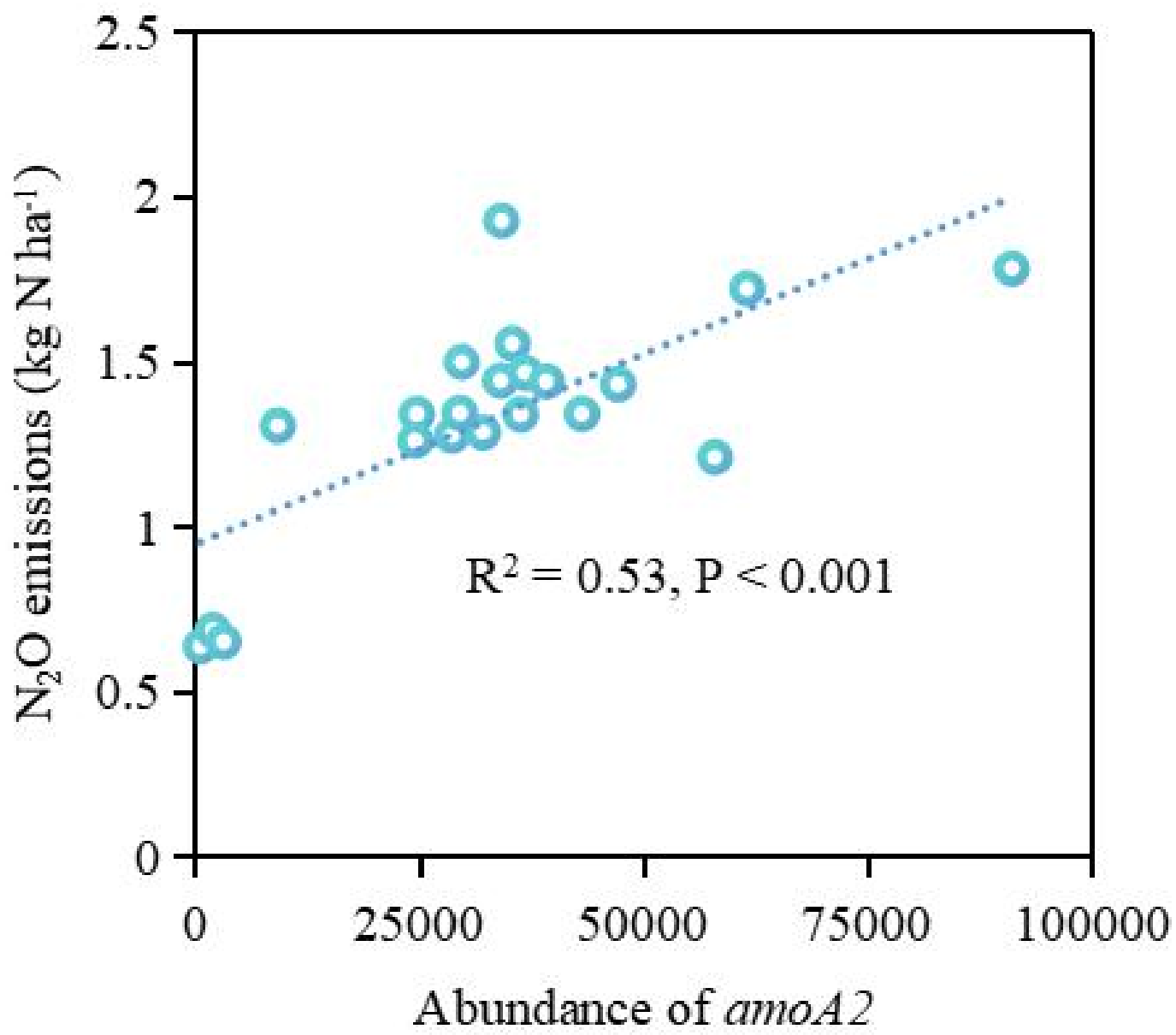

Supplementary Figure 2 Correlation between N_2O emissions and *amoA2* abundance in soil.

Supplementary Table 1 Functional genes involved in soil C and N cycling quantified in this study

No.	Classification	Gene name	Encoded protein	Functionality	Primers	
					Forward sequence	Reverse sequence
1	C degradation	*abf*A	α-L-arabinofuranosidase	Hemicellulose hydrolysis	CGSTAYCCSGGCGGCAAYTT	TGCCASGGNCCGTCCATYTC
2	C degradation	*amy*A	α-amylase	Starch hydrolysis	YGGTTTTCGTCTTGACGCSG	MGGCTGMGTRTCATGRTTK
3	C degradation	*amy*X	pullulanase	Starch hydrolysis	TATAAYTGGGGMTATGAYCC	CCCATYAAATCAAAWCGRAA
4	C degradation	*apu*	amylopullulanase	Starch hydrolysis	ACVTGGATAGGYGAGCCYCA	CCRTCSGGGAAGTAGTTKCC
5	C degradation	*cdh*	cellobiose dehydrogenase	Cellulose hydrolysis	ATWRYCTWCCGMRTHGCCMT	GTKAGSGGRTTBYKGRYCAT
6	C degradation	*cex*	exoglucanase	Cellulose hydrolysis	YSTACGGSATGCACTGGMT	TANCGCAGRTAGTCVCCCAT
7	C degradation	*chi*A	endochitinase	Chitin hydrolysis	TSAAGAARTACGCSGACAACG	ASGTCATCAGRCCCTTSAG
8	C degradation	*exo-chi*	exochitinase	Chitin hydrolysis	GATTGGTSVCAATATGAYRG	STCCARCCACCRAYRCTRAA
9	C degradation	*glx*	glyoxal oxidase	Lignin hydrolysis	AACCAGTCGATCATCTACGA	RTGSACGAGCTCDGGCATGG
10	C degradation	*iso-plu*	isopullulanase	Pullulan hydrolysis	GTCATYTACTTYGGNCC	CGNGCSACATCNGCCCA
11	C degradation	*lig*	lignin peroxidase	Lignin hydrolysis	CCGCACACACTGTTGCTGC	CGAAGGATTGCCACTCGCA
12	C degradation	*man*B	β-mannanase	Hemicellulose hydrolysis	ATGCGCGGBGTCAACCA	TCGTTGSCGATGTTGABGA
13	C degradation	*mnp*	manganese peroxidase	Lignin hydrolysis	MACRCCSTTCGACTCSACC	ACGTCSGAGCAGTCRAYGA

14	C degradation	*naglu*	α-N-acetylglucosaminidase	Cellulose hydrolysis	TVAAYTGGTAYCTGAAATAY	CCRTGYAGVGCCATCCAGTC
15	C degradation	*pgu*	pectinase/polygalacturonase	Pectin hydrolysis	ANCATTGGTGGCCSTGGAA	TTRAYGGCRATRCARTCRTC
16	C degradation	*pox*	phenol oxidase	Lignin hydrolysis	ACYAGTATCCATTGGCACGGT	AGATGVGARTGATACCARAA
17	C degradation	*sga*	glucoamylase	Starch hydrolysis	CGSAACTGGGAYTACCGS	TCCCACAGSCCSKCGTC
18	C degradation	*xyl*A	xylose isomerase	Hemicellulose hydrolysis	TGGGGBGGTCGYGAAGG	ACTTTGGCRTCRAAGTT
19	C fixation	*acc*A	acetyl-CoA carboxylase carboxyltransferase α subunit	C fixation	GAAGGCTAYCGCAARGC	CCTTCMGGSGARATMAC
20	C fixation	*acl*B	ATP-citrate lyase β subunit	C fixation	TGGACMATGGTDGCYGGKGGT	ATAGTTKGGSCCACCTCTTC
21	C fixation	*acs*A	acetyl-coenzyme A synthetase	C fixation	GATACCTGGTGGCAGACCGA	TGATCACGTCGTCGACCCGG
22	C fixation	*acs*B	acetyl-CoA synthase complex β subunit	C fixation	CTYTGYCAGTCMTTYGCBCC	CCCATAAABCCYGGDGTYTG
23	C fixation	*acs*E	5-methyltetrahydrofolate corrinoid methyltransferase	C fixation	TCATCGGCGAACGCATCAAC	AGRCCGGCTTCSATGGC
24	C fixation	*frd*A	fumarate reductase flavoprotein subunit	C fixation	MTGCTGCACACSCTGTW	CCGGTSGGGTGRWACTG
25	C fixation	*kor*A	2-oxoglutarate ferredoxin oxidoreductase α subunit	C fixation	GCCGGCTACCCCATCACCCC	ATGATGGGATGGTCGCCATG
26	C fixation	*mct*	mesaconyl-CoA C1-C4 CoA transferase	C fixation	TGGGCGCSGASGTSATMCG	TTGACSGTRTARTCSAYSGC
27	C fixation	*mcr*	malonyl-CoA reductase	C fixation	GGTGGTGTMGGDTTCACMCARTA	CGTTCATBGCGTAGTTVGGRTAGT

28	C fixation	*pcc*A	acetyl/propionyl-CoA carboxylase alpha	C fixation	GTGMTGATCAAGGCCWC	CGSGTGTTCATYTCSAGGAA
29	C fixation	*rbc*L	ribulose-bisphosphate carboxylase large chain	C fixation	AAGGAYGACGAGAACATC	TGCAGSATCATGTCRTT
30	C fixation	*smt*A	succinyl-CoA:(S)-malate CoA transferase subunit A	C fixation	TTTCTGGCCGGBTAYGCDGC	CGGTACGGHCCGGTYTGVCC
31	N Cycling	*amo*A1	ammonia monooxygenase α subunit (Archaea)	Nitrification	STAATGGTCTGGCTTAGACG	GCGGCCATCCATCTGTATGT
32	N Cycling	*amo*A2	ammonia monooxygenase α subunit (Bacteria)	Nitrification	GGGGTTTCTACTGGTGGT	CCCCTCKGSAAAGCCTTCTT
33	N Cycling	*amo*B	ammonia monooxygenase β subunit	Nitrification	TGGTAYGACATKAWATGG	RCGSGGCARGAACATSGG
34	N Cycling	*gdh*A	glutamate dehydrogenase	Organic N mineralization	GCCATCGGYCCWTACAAGGG	ATGTCRCCNGCCGGAACGTC
35	N Cycling	*hao*	Hydroxylamine oxidoreductase	Nitrification	TGTCACATGGGTGTAGACCA	ACCTGGAACATACCCAT
36	N Cycling	*hzo*	hydrazine oxidoreductase	Anaerobic ammonium oxidation	AAGACNTGYCAYTGGGGWAAA	GACATACCCATACTKGTRTANACNGT
37	N Cycling	*hzs*A	hydrazine synthase α subunit	Anaerobic ammonium oxidation	WTYGGKTATCARTATGTAG	AAABGGYGAATCATARTGGC
38	N Cycling	*hzs*B	hydrazine synthase β subunit	Anaerobic ammonium oxidation	ARGGHTGGGGHAGYTGGAAG	GTYCCHACRTCATGVGTCTG
39	N Cycling	*nap*A	periplasmic nitrate reductase	Dissimilatory N reduction	CTGGACIATGGGYTTIAACCA	CCTTCYTTYTCIACCCACAT

40	N Cycling	*nar*G	nitrate reductase α chain	Denitrification	TAYGTSGGGCAGGARAAACTG	CGTAGAAGAAGCTGGTGCTGT
41	N Cycling	*nas*A	assimilatory nitrate reductase catalytic subunit	Assimilatory N reduction	CARCCNAAYGCNATGGG	ATNGTRTGCCAYTGRTC
42	N Cycling	*nif*H	nitrogenase iron protein	N fixation	AAAGGYGGWATCGGYAARTCCACCAC	TGSGCYTTGTCYTCRCGGATBGGCAT
43	N Cycling	*nir*K1	copper-containing nitrite reductase (NO-forming)	Denitrification	GGMATGGTKCCSTGGCA	GCCTCGATCAGRTTRTGGTT
44	N Cycling	*nir*K2	copper-containing nitrite reductase (NO-forming)	Denitrification	ATGGCGCCATCATGGTNYTNCC	TCGAAGGCCTCGATNARRTTRTG
45	N Cycling	*nir*K3	copper-containing nitrite reductase (NO-forming)	Denitrification	TGCACATCGCCAACGGNATGTWYGG	GGCGCGGAAGATGSHRTGRTCNAC
46	N Cycling	*nir*S1	cytochrome cd$_1$ nitrite reductase (NO-forming)	Denitrification	GTSAACGTSAAGGARACSGG	GASTTCGGRTGSGTCTTGA
47	N Cycling	*nir*S2	cytochrome cd$_1$ nitrite reductase (NO-forming)	Denitrification	ATCGTCAACGTCAARGARACVGG	TTCGGGTGCGTCTTSABGAASAG
48	N Cycling	*nir*S3	cytochrome cd$_1$ nitrite reductase (NO-forming)	Denitrification	TGGAGAACGCCGGNCARGTNTGG	GATGATGTCCACGGCNACRTANGG
49	N Cycling	*nos*Z1	nitrous-oxide reductase	Denitrification	CGCRACGGCAASAAGGTSMSSGT	CAKRTGCAKSGCRTGGCAGAA
50	N Cycling	*nos*Z2	nitrous-oxide reductase	Denitrification	CGYTGTTCMTCGACAGCCAG	CGSACCTTSTTGCCSTYGCG
51	N Cycling	*nxr*A	nitrite oxidoreductase α subunit	Nitrification	CAGACCGACGTGTGCGAAAG	TCCACAAGGAACGGAAGGTC
52	N Cycling	*ure*C	urease α subunit	Ammonification	AAGMTSCACGAGGACTGGGG	AGRTGGTGGCASACCATSAGCAT

Supplementary Table 2 Maize yield (Mg ha^{-1}) under different fertilization treatments in 2021 and 2022

Treatment	2021	2022	Avg.
CK	5.51 (0.64)[a]	4.55 (0.35)	5.03 (0.21) b[b]
CN	9.83 (0.22)	11.58 (2.46)	10.70 (1.14) a
CNM	9.37 (1.34)	10.70 (1.21)	10.04 (0.14) a
CNS	9.38 (1.05)	11.45 (1.14)	10.41 (1.01) a
CM	11.55 (0.82)	11.25 (0.50)	11.40 (0.66) a
CT	10.77 (0.11)	11.70 (1.82)	11.24 (0.92) a
NT	10.81 (1.02)	10.64 (1.75)	10.72 (0.88) a
F and P values of fixed effects			
Treatment	$F(6, 12) = 20.24, P < 0.001$		
Year	$F(1, 14) = 3.17, P = 0.096$		
Treatment × Year	$F(6, 14) = 1.36, P = 0.295$		

[a] Numbers in brackets are standard deviations (*n* = 3).

[b] Different letters indicate Tukey-adjusted differences among treatment marginal means averaged across two years at the level of 0.05 based on linear mixed-effects model.

CK, no N fertilizer; CN, conventional urea; CNM, 75% urea N plus 25% composted poultry manure N; CNS, 75% urea N plus 25% composted slurry N; CM, 100% composted poultry manure N; CT, 75% urea N plus 25% straw N with deep tillage; NT, 75% urea N plus 25% straw N with no tillage.
